# Projection Profiling: A Data Compressing Strategy in Three-Dimensional Liquid Chromatography for Quality Control of Traditional Herbal Medicine

**DOI:** 10.3390/s25072015

**Published:** 2025-03-23

**Authors:** Jing Zhang

**Affiliations:** 1 College of Life Science and Technology, Tarim University, Alar 843300, China; zhang.jing@taru.edu.cn; 2 State Key Laboratory Incubation Base for Conservation and Utilization of Bio-Resource in Tarim Basin, Tarim University, Alar 843300, China

**Keywords:** projection profiling, baseline correction, analytical method validation, traditional herbal medicine, quality control

## Abstract

Chromatographic fingerprint technology has become the standard of quality control for traditional herbal medicines (THMs). But several issues are associated with the wavelength selection of the representative fingerprint, such as contradictory evaluation results at different wavelengths and the accurate quantification of each composition at one wavelength. These problems can be addressed by projection profiling. Projection profiling is a collection of all sample compositions at the maximum absorption wavelength after baseline correction. In this paper, eleven baseline correction algorithms are optimized by using the effective information factor (*EI*) as an indicator. The influence of different integration methods and wavelengths on analytical method validation and similarity analysis results are discussed in detail to clarify the advantages of the projection profiling. A total of 33 batches of Compound Licorice Tablets (CLTs) were used to show the influence of different wavelengths in a similarity evaluation. The results show that projection profiling is a better choice than any chromatogram at a certain wavelength, because projection profiling is more informative, accurate, and stable.

## 1. Introduction

For centuries, traditional herbal medicines (THMs) have been widely used by billions of people all over the world as natural remedies in the prevention and treatment of diseases in clinical practice and due to their reliable therapeutic efficiency [1]. However, the variability in chemical constituents and the complexity in action mechanisms are a bottleneck for their globalization and modernization. The chemical compositions of herbal materials are greatly affected by the environment and cultivation, e.g., climate, humidity, soil, harvest season, processing, and storage conditions. Given the complex chemical composition of THMs, implementing an effective quality control strategy is crucial to ensure consistency and efficacy in production applications.

Compound Licorice Tablets (CLTs) occupy a highly peculiar position within the *Chinese Pharmacopoeia* [2], where CLTs are classified as chemical drugs for quality control purposes. However, CLTs contain both one of the most widely used herbal medicines (*glycyrrhizae radix* et rhizoma) and one of the most tightly controlled substances prone to addiction (opium powder and poppy capsule extract), both of which have intricate pharmacological properties. *Glycyrrhizae radix* et rhizome is an important raw material, which is found in more than 50% of Chinese traditional prescriptions [3]. Despite annual production volumes exceeding 20 billion tablets in China [4], the quality control and generic drug consistency evaluation (GDCE) of CLTs remain elusive when using conventional chemical drug testing methodologies. In essence, CLTs are hybrid entities, consisting of both chemical drugs and TCMs. Unfortunately, TCMs lack intellectual property rights, rendering them ineligible for GDCE. But, GDCE has traditionally been limited to chemical drugs with simple compositions, rather than herbal medicines with complex formulations. As a result, the GDCE of CLTs remains an unresolved issue.

Coincidentally, the projection profiling proposed in this paper is particularly suited for extracting information from the liquid chromatography fingerprints of complex compositions. This technique can be used for preprocessing liquid chromatography data prior to GDCE, thus addressing this challenge. Therefore, this paper uses CLTs as a case study to demonstrate the impact of chromatographic wavelength variations on method validation and similarity analysis using traditional data processing methods. Additionally, by comparing traditional data processing methods with projection profiling, the accuracy and stability of the latter are demonstrated.

At present, chromatographic fingerprint technology is widely recognized globally as an effective technique for the identification and qualification of medicinal herbs. It has also become an alternative method for evaluating the quality of herbs and their preparations [5]. A fingerprint is a characteristic profile that chemically represents the composition of a sample, typically reflecting as much information as possible. High-performance liquid chromatography with a diode array detector (HPLC-DAD) is the most widely used and well-established detection method in the application of fingerprint technology for the quality assessment of traditional Chinese medicines (TCMs). In the *Chinese Pharmacopoeia*, HPLC serves as the core method for the quality control of nearly all TCMs. Furthermore, a DAD is the most cost-effective detector that can be used in conjunction with HPLC. This is because DADs can simultaneously detect all wavelengths of ultraviolet light transmitted through the sample. In other words, DADs can produce chromatograms at any wavelength (time vs. response value), generate spectra at any time point (wavelength vs. response value), and even carry out three-dimensional chromatography (time vs. wavelength vs. response value).

However, two major issues have arisen in the process of applying GDCE using DAD raw data: data wastage and the variation in wavelength selection on the evaluation. First, a significant amount of data is not fully utilized during analysis. Although DADs can generate three-dimensional (3D) data, the sheer volume is enormous and difficult to manage. Additionally, no instrument manufacturer currently provides functionality within their HPLC workstations or software to process DAD raw data. Worse still, the volume of DAD data increases exponentially during quality control or GDCE processes. Traditionally, data processors rely on experience to select a chromatogram at a representative wavelength for analysis. While this simplifies the problem, it discards over 99% of the DAD raw data, rendering the evaluation results unreliable. Second, the evaluation results vary depending on the chosen wavelength. Different wavelengths produce different chromatograms, and a chromatogram at any single wavelength cannot fully reflect the entire sample composition. In some cases, the evaluation results for the same sample at different wavelengths can be completely opposite. This contradiction in results poses a significant challenge for quality control and GDCE.

Therefore, I propose using projection profiling to extract the maximum information from the raw data generated by HPLC-DAD. It is worth noting that detectors such as UV–Vis and MS cannot produce 3D data, and thus projection profiling cannot be applied to their data. Projection profiling employs a series of models or functions, including two-dimensional baseline correction, peak detection, peak fitting, and projection, to generate a stable and accurate profile that maximizes information from the original 3D data produced by DADs. Two-dimensional baseline correction reduces data noise caused by solvents, revealing chromatographic peaks that might be obscured by solvents, thus enhancing the information of some peaks [6]. Peak detection and peak fitting improve the automation of data processing and make peak area measurements more accurate, eliminating errors introduced by manual integration. The projection function ensures the uniqueness of the profiling while also maximizing data compression.

In summary, projection profiling utilizes the raw data from DADs, applying a series of models and functions to compress the data and generate accurate and stable profiles for quality control or GDCE of TCMs. The main purpose of this paper is to prove that projection profiling is more accurate, stable, and informative compared with a chromatogram at any wavelength and to find out the best two-dimensional baseline correction methods and to optimize their parameters for projection profiling.

## 2. Theory

### 2.1. Baseline Correction

Baseline correction is a crucial and indispensable processing step for projection profiling. Potential peak information shadowed by solvent signals at low wavelength is extracted through baseline correction. Baseline correction can generally be divided into manual, semi-automated (parametric), and fully automatic (nonparametric) methods [7]. Manual baseline correction is not the focus of this research, because of inconvenience for large scale datasets and bias towards user experience, noise levels, and baseline characteristics. Parametric methods are time-consuming when optimizing parameters. Nonparametric methods, although more convenient to use, tend to be less accurate than parametric methods [7,8]. I collected 11 open-source baseline correction methods and applied them to two-dimensional data generated by HPLC-DAD (see Table 1). These baseline correction algorithms were implemented in MATLAB (Version 2014a) on a laptop with an Intel^®^ Core^®^ i7-2860QM 2.5 GHz CPU and 16 GB of RAM, running on a Windows 10 professional operating system. The related MATLAB codes are also contained in the Supplementary Materials.

Some of the most used parametric methods, including airPLS, ALS, arPLS, and MPLS, are based on the penalized least squares (PLS, also called ALS smoother). PLS is a versatile smoothing method, which was originally proposed as ALS by [17] in 1922 and later extended by Eilers to signal smoothing [18], peak aligning [19], and baseline correction (ALS) [20]. In PLS, a trade-off parameter (λ) is used to balance between the fidelity of the original signal (evaluated by least squares curve fitting) and the roughness of the estimated baseline (penalized by n-order differences function). The airPLS method functions by iteratively adjusting the weights of the sum of squared errors (SSEs) between the baseline and the signal, determined adaptively based on the difference between them [21]. Compared with PLS, the improvement of airPLS is that the weights of different baseline region set different values obtained by a logical function. In ALS, the baseline approximation employs weights determined by the sign differences between smoothed and measured signals, with all asymmetry parameters being uniform within the pure baseline region [20]. arPLS, similar to airPLS and ALS, assigns weights differently and adds a penalty to control baseline smoothness [22]. In MPLS, the rough background is fitted using a morphological opening operation [15].

There are three other parameter methods. The ATEB combines two-side exponential smoothing strategy and iterative fitting process to gradually approximate the desired baseline [9]. Backcor is an iterative method that estimates the background using low-order polynomial fitting, with polynomial coefficients determined by minimizing a non-quadratic cost function [10]. Different cost functions are designed to minimize the influence of peaks on the estimation. BEADS decomposes chromatogram data into modeled components with the baseline and peaks [11].

The four nonparametric methods take little time to optimize the parameter (no need to even optimize). In CC, the corner points are omitted from data by an iterative manner combined with key point interpolation. Subsequently, a smooth baseline is fitted on the remaining points using the Bezier method [12]. The LMV-RSA is divided into three steps. (1) The local minimum values are detected and organized as a baseline vector. (2) Iterative optimization is employed to detect outliers by a robust statistical strategy. (3) These outlier points are estimated using linear interpolation and update the outliers until convergence [14]. The doPF is a variant of polynomial fitting algorithm. The related code was found at a part of the Finnee [23] toolbox. The SWiMA is a fully automated and model-free baseline correction method. It iteratively applies a small, but increasing, moving average window in conjunction with peak stripping to estimate baseline [16].

### 2.2. Peak Detection and Peak Integration

A typical chromatogram from a THM extract can contain hundreds of peaks, each representing different chemical components. These peaks vary in size and shape, providing detailed information about the composition of the extract. However, for the quality control of THM, some major and stable characteristic peaks need to be extracted from those complicated peaks by robust peak detection and peak integration methods [24]. In this study, the peak detection scripts were developed and modified based on the function of “findpeaksplot.m” provided by Prof. Tom O’Haver (https://terpconnect.umd.edu/~toh/spectrum/findpeaksplot.m) (accessed on 11 March 2025). The downward zero-crossing points in the first derivatives of the projection profiling were used to detect the borders of individual peaks. At the same time, strict peak detection parameters were used to ensure the reliability of the desired characteristic peaks, including slope, amplitude, peak height, peak width, and peak area. To minimize noise interference during peak detection, a pseudo-Gaussian smoothing method was applied. This method involves three passes of an unweighted moving average filter to effectively filter out noise before detecting the peaks. But to avoid distortion to the quantitation by the filter, this filtering method only worked during detection, not integration. Each peak detection condition in this article is set to the same value.

Four auto-integration methods were compared through analytical method validation in this research using manual integration as a reference. The perpendicular drop method (PD) integrates the area enclosed by the signal curve, projection, and baseline. The tangent skim method (TS, also called valley–valley method) integrates each peak at the valley between the peaks. These two integration methods can be realized by the “measurepeaks.m” function created by Prof. Tom O’Haver (http://terpconnect.umd.edu/~toh/spectrum/measurepeaks.m) (accessed on 11 March 2025). The most accurate method for measuring each peak area is to deconvolute the peaks using a fitted peak model. The fitted approach allows for the precise separation and quantification of overlapping peaks. Unfortunately, no model is fully applicable to the peak obtained from the experiment. The shape of each peak in a chromatogram may be different. The Gaussian fitting method (Equation (1)) is simple and idealized. Symmetrical chromatographic peaks are well described by the Gaussian function [25]. However, this method may overestimate the areas of sharp peaks while underestimating the areas of broadened peaks. This limitation can lead to inaccuracies in the quantification of certain components. The Gaussian fit method was realized by the “findpeaksG.m” function (http://terpconnect.umd.edu/~toh/spectrum/findpeaksG.m) (accessed on 11 March 2025).

The EMG fitting method (Equation (2)) offers greater accuracy for low-asymmetric peaks [26]. On the other hand, the EGH method is mathematically simple, numerically stable, and its related open-source code is available on GitHub (https://github.com/chemplexity/chromatography/tree/master/Methods/Integration/ExponentialGaussian.m) (accessed on 11 March 2025).

(1) $$f_{Gaussian}(t)=H\;\exp(\frac{-(t-t_{R}{)}^{2}}{2\sigma^{2}})$$

(2) $$f_{EGH}(t)\equiv \left\{\begin{array}{c} H\exp(\frac{-(t-t_{R}{)}^{2}}{2\sigma_{g}^{2}+\tau (t-t_{R})})\\ 0 \end{array}\right.\begin{array}{c} ,2\sigma_{g}^{2}+\tau (t-t_{R})>0\\ ,2\sigma_{g}^{2}+\tau (t-t_{R})\leq 0 \end{array}$$

where *H* is the height of the peak maximum, *t_R_* is the retention time of the peak maximum, *σ_g_* is the standard deviation of the peak, and *τ* is the time constant of exponential decay.

### 2.3. Similarity Analysis

The similarity analysis of chromatographic herbal fingerprints has become a widely used tool in the quality assessment of THM. It plays a crucial role in ensuring consistency and identifying potential variations in herbal formulations. And some parameters have been applied to express the (dis)similarity between two fingerprints in quality, quantity, and variation. In this paper, three qualitative parameters (*S_F_*, Equation (3); *S_F_’*, Equation (4); *S_R_*, Equation (5)) and three quantitative parameters (*L*, Equation (6); *M*, Equation (7); *R*, Equation (8)) were used to evaluate the projection profiling and the chromatograms at all wavelengths of the 33 batches of CLTs. These parameters show different biases to different peaks [6,27]. For example, *S_F_* and *L* were biased to strong peaks; *S_F_’* and *M* were biased to weak peaks; and *S_R_* and *R* had no obvious bias. The main idea of similarity analysis is to regard the peak areas of the fingerprints after the peak matching as a vector. Then, the similarity between fingerprints is converted into the similarity between vectors. So, the sample fingerprint (SFP) vector is defined as $\vec{x}=\left(x_{1},x_{2},\ldots x_{n}\right)$. Correspondingly, the reference fingerprint (RFP) vector is expressed by $\vec{y}=\left(y_{1},y_{2},\ldots y_{n}\right)$, where *x_i_* and *y_i_* are the peak areas in the sample fingerprint and reference fingerprint, respectively. The RFP is generated by the median method to avoid the influence of outliers. *S_F_* is the cosine of the angle of $\vec{x}\vec{x}$ and $\vec{y}\vec{y}$. To increase the weight of weak peaks, $\vec{x}$ is transformed into $\vec{r}=\left(r_{1,}r_{2},\ldots r_{n}\right)$ or $\vec{p}=\left(p_{1,}p_{2},\ldots p_{n}\right)$, where *r_i_* is equal to *x_i_*/*y_i_* and *p_i_* is equal to *x_i_*/*y_i_* when *x_i_* < *y_i_* and to 2-*y_i_*/*x_i_* when *x_i_* ≥ *y_i_*. Then, $\vec{y}$ is transformed into $\vec{u}=\left(u_{1,}u_{2},\ldots u_{n}\right)$, as an n-dimensional unit vector. *S_F_’* is the cosine of the angle of $\vec{r}$ and $\vec{u}$. *S_R_* is the cosine of the angle of $\vec{p}$ and $\vec{u}$. *L* is the ratio of the norm of $\vec{x}$ and $\vec{y}$. *M* is the mean of the corresponding peak area ratio of SFP and RFP. *R* is the ratio of the total peak areas of SFP and RFP. The vector norm is written with a double bar.

(3) $$S_{F}=cos(∠\vec{x}\vec{y})=\frac{\vec{x}\cdot \vec{y}}{\left\|\vec{x}\right\|\left\|\vec{y}\right\|}=\frac{\sum_{i=1}^{n}x_{i}y_{i}}{\sqrt{\sum_{i=1}^{n}{x_{i}}^{2}}\sqrt{\sum_{i=1}^{n}{y_{i}}^{2}}}$$

(4) $${S_{F}}^{\prime }=cos(∠\vec{r}\vec{u})=\frac{\vec{r}\cdot \vec{u}}{\left\|\vec{r}\right\|\left\|\vec{u}\right\|}=\frac{\sum_{i=1}^{n}\frac{x_{i}}{y_{i}}}{\sqrt{n\sum_{i=1}^{n}(\frac{x_{i}}{y_{i}}{)}^{2}}}$$

(5) $$S_{R}=cos(∠\vec{p}\vec{u})=\frac{\vec{p}\cdot \vec{u}}{\left\|\vec{p}\right\|\left\|\vec{u}\right\|}=\frac{\sum_{i=1}^{n}p_{i}}{\sqrt{n\sum_{i=1}^{n}{p_{i}}^{2}}}$$

(6) $$L=\frac{\left\|\vec{x}\right\|}{\left\|\vec{y}\right\|}=\frac{\sqrt{\sum_{i=1}^{n}{x_{i}}^{2}}}{\sqrt{\sum_{i=1}^{n}{y_{i}}^{2}}}\times 100\%$$

(7) $$M=\frac{1}{n}\sum_{i=1}^{n}\frac{x_{i}}{y_{i}}\times 100\%$$

(8) $$R=\frac{\sum_{i=1}^{n}x_{i}}{\sum_{i=1}^{n}y_{i}}\times 100\%=\frac{\bar{x}}{\bar{y}}\times 100\%$$

## 3. Materials and Methods

### 3.1. Materials and Reagents

All CLTs (labeled S1–S33, see Table S1 for production batch information) were obtained from the same manufacturer, Guizhou Guangzheng Pharmaceutical Co., Ltd. (Guiyang, China). Reference standards of morphine (MPE, purity > 99.8%), liquiritin (LQT, purity > 93.1%), codeine phosphate (CON, purity > 97.5%), sodium benzoate (SMB, purity > 99.7%), and glycyrrhizic acid (GLA, purity > 98.0%) were acquired from the National Institutes for Food and Drug Control(Beijing, China). The chemical structures of five reference standards are shown in Figure S1. The herbal materials that comprise the CLTs are listed in Table S2.

Acetonitrile and methanol (both HPLC-grade) were supplied from Yuwang Chemical Industry Co., Ltd. (Shandong, China). Phosphoric acid (HPLC-grade) and sodium 1-heptanesulfonate (HPLC-grade) were sourced from Kelong Chemical Reagent Factory (Chengdu, China) and Zhongmei Chromatographic Co., Ltd. (Shandong, China), respectively. All other chemicals were analytical-grade, and deionized water was purified using a Milli-Q system (Bedford, MA, USA).

### 3.2. Standards and Sample Preparation

The reference standard solution was prepared by accurately weighing 1 mg of MPE, LQT, CON, SMB, and GLA separately, and then dissolving each in a 10 mL volumetric flask using a methanol–water–phosphoric acid mixture (160:40:1, *v*:*v*). For CLT solutions, 20 tablets were milled, and the powder of 4 tablets was weighed (1.5 g per tablet) and transferred to a 50 mL flask. The powder was extracted with 50 mL of the methanol–water–phosphoric acid mixture in an ultrasonic bath at 45 °C, 240 W, and 40 kHz for 10 min. All solutions were filtered through 0.45 μm Millipore filters and stored at 4 °C in the dark prior to analysis.

### 3.3. HPLC Analysis

All analysis was performed on a reverse phase-HPLC Agilent 1100 system, equipped with a micro-vacuum degasser (G1379A), a quaternary pump (G1311A), an automatic sampler (G1313A), a diode array detector (DAD, G1315B), and a system controller linked to an Agilent OpenLAB CDS ChemStation data handling system (Edition C.01.07 SR3). Separations were carried out on a COSMOSIL C18 column (5 μm, 250 mm × 4.6 mm i.d., Nacalai, Kyoto, Japan).

The mobile phase was composed of aqueous solution acidified with 0.2% phosphoric acid (Solvent A, and containing 5 mM sodium 1-heptanesulfonate) and acetonitrile–methanol (Solvent B, 9:1, *v*:*v*). The gradient elution program was as follows: 4–21% B at 0–10 min; 21–35% B at 10–20 min; 35–53% B at 20–32 min; 53–82% B at 32–45 min; and 82–4% B at 45–50 min. The column temperature was kept at 35 °C. The injection volume was 5 μL. The flow rate was 1 mL/min. The representative chromatogram was empirically selected at 220 nm. The DAD detection was conducted from 190 to 600 nm, with a sampling frequency of 2.5 Hz.

## 4. Results and Discussion

To effectively convey the core concepts of this article, I briefly introduce the purpose and rationale behind each subsection, as well as the development of projection profiling. Projection profiling is a data compression and extraction technique specifically designed for DAD raw data. It applies baseline correction to subtract the solvent response at low wavelengths and uses projection to uncover obscured chromatographic peaks. Traditionally, chromatographic data processing begins with wavelength selection, a step that relies on the data processor’s expertise and can introduce errors that often go unnoticed. In Section 4.1, I present evidence demonstrating the impact of wavelength selection on method validation. Section 4.4 further explores its influence on similarity assessments in quality control and GDCE. During the projection process, no manipulation occurs at the maximum absorption wavelength of each chromatographic peak. Instead, projection profiling segments and reassembles the data at these wavelengths, ensuring that no peak data are altered. This approach maximizes information content, reduces redundancy, and preserves data integrity. These concepts are further discussed in Section 4.2. As the first step in projection profiling involves the baseline correction of three-dimensional data, I compared the 11 most widely used baseline correction algorithms to evaluate their suitability for this technique. This comparison is detailed in Section 4.3.

### 4.1. Effects of Wavelength and Integration on Analytical Method Validation

Analytical method validation is the process of ensuring that an analytical procedure is suitable for its intended purpose. It also confirms the method’s reliability and acceptance for accurate and consistent results. In this process, qualified optimal operating parameters settings are critical to the analysis, e.g., detector settings (selection of representative chromatograms), peak detection parameters, and integration methods. However, the selection of a representative chromatogram at a suitable wavelength is difficult and ambiguous for such a complex system as THM. Because as the wavelength changes, the response of each component will change to varying degrees. Unrepresentative chromatograms will have serious bias in the final evaluation results. Animation S1 (a GIF file in the Supplementary Materials) intuitively shows the integration results of CLT at different wavelengths (190–410 nm) under the same integration conditions.

The peak area measurement, including peak detection and peak integration, is a crucial step for accurate quantification. Since the retention times of the five standards are known, peak detection was not involved here. Different methods or parameters in the measurement of peak area affects the evaluation results. But how much does integration and wavelength affect analytical method validation and final evaluation results? The in-depth and detailed analysis and discussion are outlined in this Section and in Section 4.4.

Of the typical validation characteristics, precision (repeatability), linearity, and accuracy have the most direct and significant relationship with quantification. Therefore, this paper discussed the effects of wavelength and integration on analytical method validation from these three aspects.

#### 4.1.1. Precision (Repeatability)

**Figure 1:**
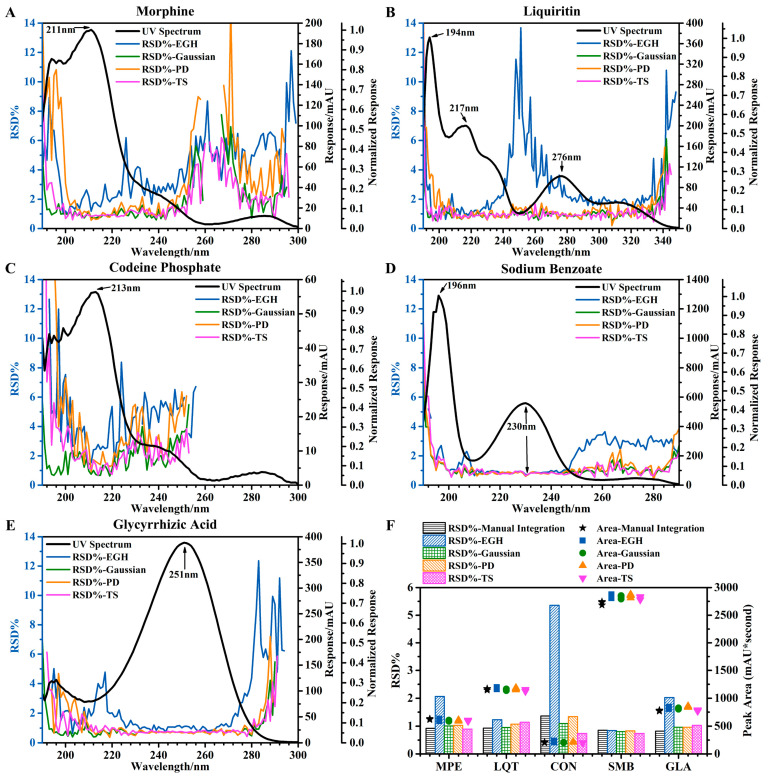
Effects of different wavelengths and different integration methods on precision. (**A**–**E**) Trends of RSD of the five standards using four integration methods at different wavelengths. (**F**) Precision of five standards at 220 nm evaluated by four integration methods and manual integration.

Repeatability was assessed using six consecutive determinations of mixed standard solution at 100 percent of the sample test concentration. To visually display a large amount of data and information, the trends of relative standard deviation (RSD) of the five standards using four integration methods at different wavelengths are shown in five graphs (Figure 1A–E) with three *y*-axes (RSD%, response, and normalized response). Normalization (Equation (9)) rescales the responses of different standards into the same range of [0, 1]. This might be useful for the comparison between standards. Because the response is always positive or sometimes somewhat negative and its minimum is close to zero, the formula was further simplified.

(9) $${X_{i}}^{\prime }=\frac{X_{i}-Min(X)}{Max(X)-Min(X)}\approx \frac{X_{i}}{Max(X)}$$

where *Xi* is the origin response and *Xi′* is the normalized response. On the whole, integration method and wavelength (including response and normalized response) affected the repeatability to varying degrees. The ability to influence is as follows: integration method > wavelength (response > normalized response).

For the integration methods, the wavelengths were compared (horizontal comparison), and the smaller the “fluctuation” of the RSD curve, the more “robust” the integration method. Robustness: EGH ≪ PD < TS ≈ Gaussian. At the same time, when comparing the integration methods (vertical comparison), the lower the RSD, the better “the precision of integration method” (based on good robustness). The precision of the integration method: EGH < PD < TS ≈ Gaussian.

When the value of normalized response was less than 0.05 (a critical point), the robustness and precision of the integration method were significantly reduced, which were reflected in the significant increase in the fluctuation of the RSD curve and the value of RSD, respectively. It should be noted in particular that when the value of the normalized response was less than 0.1, the precision evaluated by EGH was greatly affected (Figure 1B). This critical point reflected well in Figure 1A: at 254–277 nm (normalized response < 0.05), the RSD increased significantly. Meanwhile, at 277–291 nm (normalized response > 0.05), except for EGH, the RSD obtained by the other three integration methods declined. Critical points also appeared in Figure 1B (at 334–350 nm), Figure 1D (at 250–290 nm), and Figure 1E (at 283–300 nm).

When the value of response was too small (less than about 30 mAU), the fluctuation of the RSD curve increased significantly (Figure 1A at 242–300 nm; Figure 1C at 222–250 nm). The small response was even difficult to accurately integrate, as shown in Figure 1C at 260–300 nm. In the low wavelength range (190–205 nm) due to the influence of solvents (methanol, acetonitrile), the results were unreliable and unacceptable. Also, we compared four integration methods (using manual integration as a reference) in evaluating the precision of five standards at 220 nm (Figure 1F). The peak area measurement by TS was slightly lower than the other four methods. The RSD obtained by EGH was significantly affected by the peak area and was inversely proportional to it. Except for EGH, other integration methods obtained similar results, including peak area and RSD.

Finally, I concluded that the RSD of repeatability for five standards is approximately equal to 1% under certain conditions and ranges: response > 30 mAU, normalized response > 0.05, integration method except EGH, and wavelength except 190–205 nm. Notice that the conditions of response and normalized response can be met by choosing an appropriate wavelength.

#### 4.1.2. Linearity

**Figure 2:**
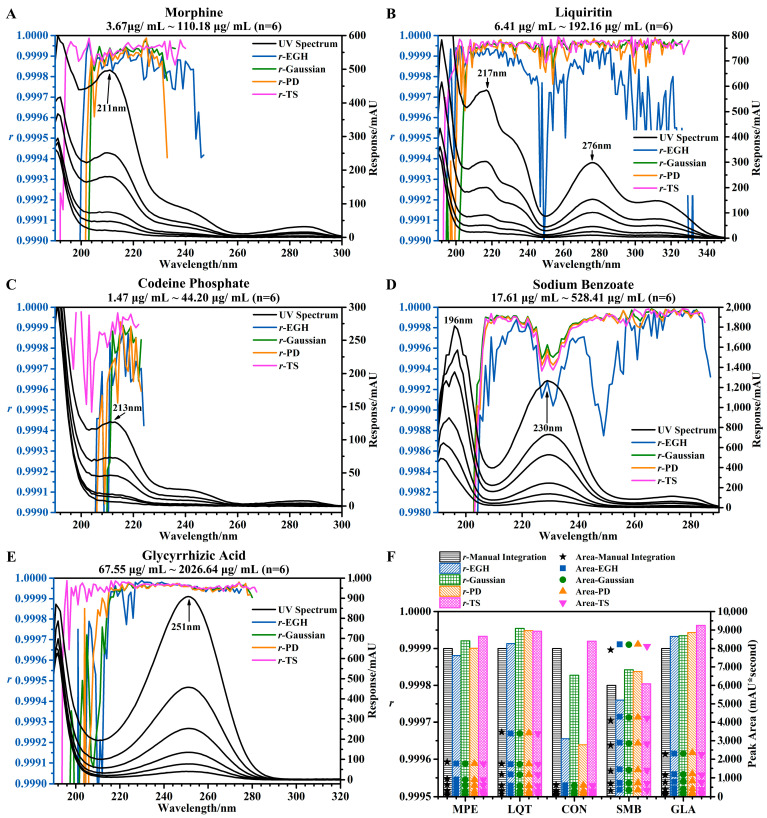
Effects of different wavelengths and different integration methods on linearity. (**A**–**E**) UV spectra of the five standards at six different concentrations and the trends of r of the five standards using four integration methods at different wavelengths. (**F**) Linearity of five standards at 220 nm evaluated by four integration methods and manual integration.

The linearity was evaluated across the range (at 10, 25, 50, 100, 150, and 300 percent, n = 6) of the sample test concentrations by the dilution of five standards of stock solution. The linearity was determined by the calculation of a regression line via the method of least squares, *y* = *a* + *bx*, namely A = *a* + *b*C. Calibration curves were constructed by plotting the peak area (A, mAU) on the *y*-axis against the concentration (C, μg/mL) of the five compounds on the *x*-axis. The correlation coefficient (*r*, Equation (10)), y-intercept (*a*, Equation (11)), and slope of the regression line (*b*, Equation (12)) were used to provide mathematical estimates of the degree of linearity for the constructed calibration curves.

(10) $$r=\frac{\sum_{i=1}^{n}\left(x_{i}-\bar{x})(y_{i}-\bar{y}\right)}{\sqrt{\sum_{i=1}^{n}(x_{i}-\bar{x}{)}^{2}}\sqrt{\sum_{i=1}^{n}(y_{i}-\bar{y}{)}^{2}}}=\frac{\sum_{i=1}^{n}x_{i}y_{i}-n\bar{x}\cdot \bar{y}}{\sqrt{(\sum_{i=1}^{n}{x_{i}}^{2}-n{\bar{x}}^{2})}\sqrt{(\sum_{i=1}^{n}{y_{i}}^{2}-n{\bar{y}}^{2})}}$$

(11) $$a=\bar{y}-b\bar{x}=\frac{\bar{y}(\sum_{i=1}^{n}{x_{i}}^{2})-\bar{x}\sum_{i=1}^{n}x_{i}y_{i}}{\sum_{i=1}^{n}{x_{i}}^{2}-n{\bar{x}}^{2}}=\frac{\sum_{i=1}^{n}y_{i}\sum_{i=1}^{n}{x_{i}}^{2}-\sum_{i=1}^{n}x_{i}\sum_{i=1}^{n}x_{i}y_{i}}{n\sum_{i=1}^{n}{x_{i}}^{2}-(\sum_{i=1}^{n}x_{i}{)}^{2}}$$

(12) $$b=\frac{\sum_{i=1}^{n}\left(x_{i}-\bar{x})(y_{i}-\bar{y}\right)}{\sum_{i=1}^{n}(x_{i}-\bar{x}{)}^{2}}=\frac{(\sum_{i=1}^{n}x_{i}y_{i})-n\bar{x}\cdot \bar{y}}{\sum_{i=1}^{n}{x_{i}}^{2}-n{\bar{x}}^{2}}=\frac{n\sum_{i=1}^{n}x_{i}y_{i}-\sum_{i=1}^{n}x_{i}\sum_{i=1}^{n}y_{i}}{n\sum_{i=1}^{n}{x_{i}}^{2}-(\sum_{i=1}^{n}x_{i}{)}^{2}}$$

where x is the peak area, y is the concentration, and n is the number of determinations. It is worth noting that the three parameters (r, a, and b) obtained by different integration methods at different wavelengths were a little different, and b was applied for the calculation of accuracy (Section 4.1.3). The UV spectra of the five standards at six different concentrations and the trends of r of the five standards using four integration methods at different wavelengths are shown in Figure 2A–E.

In this linear range, the response at the lowest concentration (10%) should not be less than 3.3 mAU. In other words, the response at the highest concentration (300%) should not be less than 100 mAU. Otherwise, it was impossible to accurately measure the peak area, e.g., Figure 2A at 240–300 nm, Figure 2C at 224–300 nm, and Figure 2E at 280–320 nm. And the reliability of the correlation coefficient was based on the accurate integration of small peaks. However, this was difficult to accomplish with EGH (Figure 1B,D and Figure 2B,D).

Similarly, suitable wavelengths should not fall in the range affected by solvent noise (from 190 to 205 nm or even 210 nm). TS was more resistant to the solvent noise than other integration methods (Figure 2A,C,E). In general, a higher response was beneficial for obtaining a stable correlation coefficient. It is interesting that in some special cases, the higher the response, the lower the r (Figure 2D at 223–230 nm). This may be due to an excessive concentration range. But this has little effect on the r. I also compared four integration methods (using manual integration as a reference) in evaluating the linearity of five standards at 220 nm (Figure 2F). The difference in r was a minimal between the four integration methods, except that a slightly smaller r was obtained by EGH and PD for standard CON. The order of the r values obtained by different integration methods was as follows: TS > Gaussian > manual integration > PD > EGH.

Therefore, I summarized the following: under certain conditions and ranges (a response of 300%, a concentration higher than 100 mAU, the integration method except for EGH, and a wavelength, except 190–210 nm), the *r* was more than 0.9998 in most cases. In some cases (inappropriate concentration range), the *r* was still higher than 0.9994, such as in Figure 2D at 223–238 nm and Figure 2C at 210–225 nm.

#### 4.1.3. Accuracy

**Figure 3:**
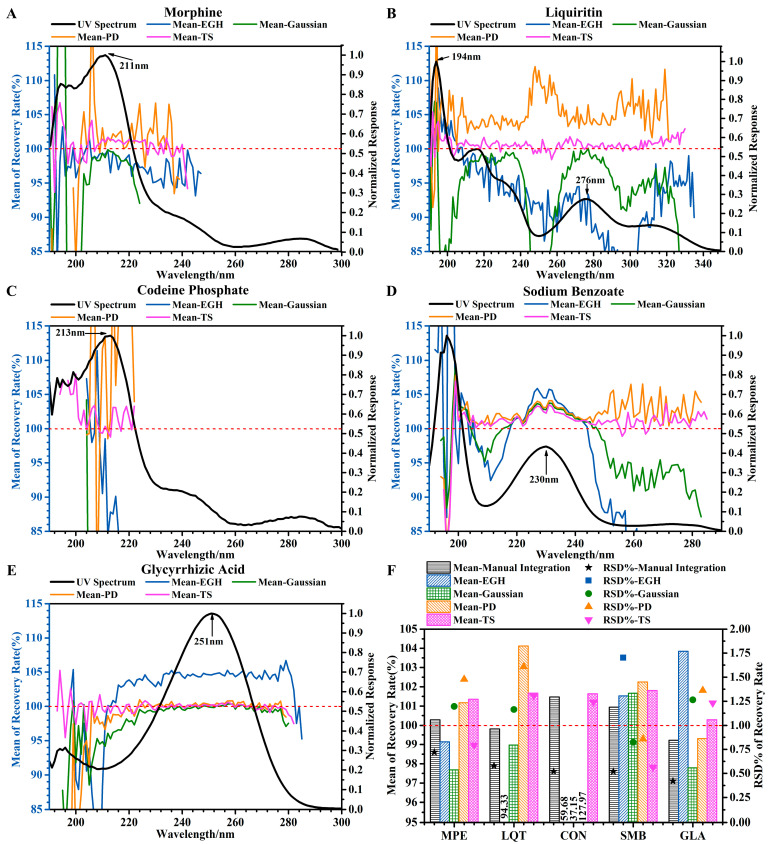
Effects of different wavelengths and different integration methods on accuracy. (**A**–**E**) Trends of mean recovery (%) of the five standards using four integration methods at different wavelengths. (**F**) Accuracy of five standards at 220 nm evaluated by four integration methods and manual integration. The red dotted line is intended to facilitate readers in comparing it with the value of 100% on the vertical axis.

Accuracy was assessed using six determinations at 100 percent of the sample test concentration by spiking the five standard solutions. The recovery was calculated using Equation (13).

(13) $$Recovery(\%)=\frac{C_{spiked\;sample}-C_{unspiked\;sample}}{C_{control\;spike}}\times 100\%\;=\frac{{A_{i}}_{\;(spiked\;sample)}-\frac{m_{i}}{m}A_{unspiked\;sample}}{C_{control\;spike}\times b}\times 100\%$$

where *A_i_*_(*spiked sample*)_ is obtained by linear regression equation, *A* = *a* + *bC*; *m_i_* is the spiked sample weight of the *i*th determination; and *m* is the unspiked sample weight. The trends of mean recovery (%) of the five standards using four integration methods at different wavelengths are shown in Figure 3A–E. For integration methods, only TS was stable and acceptable. The significant errors were found by the other three integration methods in the accuracy of evaluation. This may be due to the low robustness and stability of these integration methods, e.g., the recovery was significantly overestimated by PD and was significantly underestimated by Gaussian and EGH in Figure 3B at full wavelength. On the contrary, the recovery was significantly overestimated by EGH in Figure 3E at 210–280 nm.

Comparing with manual integration (Figure 3F), the mean of recovery (100–102%) and the RSD% of recovery (less than 1.5%) obtained by TS were slightly higher. The accuracy should be based on good linearity and precision. Therefore, under the conditions of linearity and precision, the mean recovery obtained by the TS was 98–103% for the five standards.

In a word, the effects of wavelength and integration on the analytical method validation cannot be ignored. For integration methods, only TS met the expected requirements. And for wavelength selection, a wide range of wavelengths (which met a certain required response or normalized response, except 190–210 nm) can be accepted as a single standard. Principally, the higher the response and normalized response, the more reliable the results.

However, for THMs containing complex components, the range of wavelengths was limited and difficult to determine. Therefore, I proposed replacing the chromatograms at certain wavelengths with projection profiling to obtain the highest response for the quality control of THM.

### 4.2. Chromatogram vs. Projection Profiling

**Figure 4:**
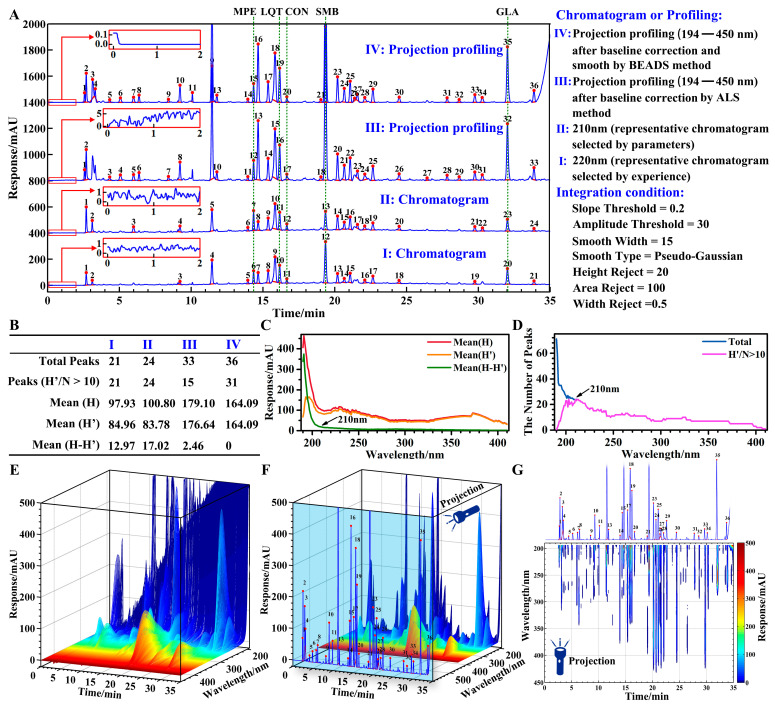
Comparison of chromatogram and projection profiling. (**A**) Representative chromatograms and projection profiles (Some of the baselines are enlarged and displayed within the red box.). (**B**) Chromatographic information parameters. (**C**) Trends of average baseline heights at different wavelengths. (**D**) Trends of the number of quantifiable peaks at different wavelengths. (**E**) Original 3D image of CLT. (**F**) Three-dimensional image after baseline correction of CLT. (**G**) Aerial view after baseline correction of CLT.

The routine method of selecting a representative chromatogram based on experience is easy and convenient, but ambiguous and not convincing. In this paper, representative chromatograms were selected by experience at 220 nm (Figure 4(A-I)). However, to explain the selection process more scientifically and reasonably, I proposed the selection of a representative chromatogram by two parameters reflecting chromatographic information.

The first parameter is the average baseline height (Mean(H-H′)). It was used to assess the extent of baseline drift. A value of the average baseline height that is closer to zero shows a slighter drift in the baseline. Where H is the response of the peak apex; H′ is the response difference between the peak apex and the peak valley. The trends of the average baseline height at different wavelengths are shown in Figure 4C.

The second parameter is the number of quantifiable peaks (Peaks(H′/N > 10)). It was used to evaluate the amount of information. The larger the value, the greater the amount of information. And the value of the number of quantifiable peaks that is closer to total peaks shows a lower impact of baseline noise. Where N is the noise fluctuation of the baseline in the selected time ranges. In this paper, the time ranges were selected for noise assessment at 1–2 min (300 data points), 10.5–11 min (75 data points), and 32.5–33 min (75 data points). The trends of the number of quantifiable peaks at different wavelengths are shown in Figure 4D.

The chromatogram at 210 nm (Figure 4(A-II)) was selected by parameters as the representative chromatogram, due to the lower average baseline height (Figure 4C) and the higher number of quantifiable peaks (Figure 4D). In the range of 190–210 nm, the lower the wavelength, the more serious the baseline drift, the more the total peaks, but the less the quantifiable peaks (Figure 4C,D). This phenomenon indicated that baseline drift and noise may obscure a lot of useful information.

Projection profiling resolved these problems of baseline drift and noise through baseline correction (for 3D image before baseline correction see Figure 4E; for 3D image after baseline correction see Figure 4F) and obtained more chromatographic information through projection (for 3D image see Figure 4F; for aerial view see Figure 4G). The chromatograms at 190–193 nm (Figures S2–S5) were discarded due to excessive noise and interference with the projection. The BEADS method (Figure 4(A-IV)) obtained more quantifiable peaks than the ALS method (Figure 4(A-III)), because the baseline is modeled as a low-pass signal in the BEADS method [14]. The ALS method can be used not only for baseline correction but also for curve smoothing by modifying parameters appropriately. Generally, 10^−9^ ≤ *p* ≤ 10^−1^ and 10^2^ ≤ λ ≤ 10^9^ are good choices for baseline correction; 0 < *p* < 1 and 0 < λ ≤ 10^4^ are appropriate for curve smoothing [25,28]. But in the one-signal process, the ALS method only performed baseline correction or curve smoothing.

A lot of noise was contained in the projection profiling, so proper smoothing may be necessary. A smooth baseline is essential for the accurate quantification of small peaks. For example, the quantifiable peaks in the projection profiling (Figure 4(A-III)) affected by noise were less than the chromatograms at 210 and 220 nm (Figure 4B). Compared with the chromatograms, the Mean(H′) obtained from the projection profiles increased by about 100% and the total peaks increased by about 30% (Figure 4B). This shows that the projection profiling can indeed increase the amount of information.

The methodological comparison of projection profiles obtained by 10 baseline correction algorithms under optimal conditions (see Section 4.3) is shown in Figure S6. The process of analytical method validation is described in detail in Section 4.1. The TS method was adopted as the integration method to obtain more accurate and stable results. I did not further analyze the CC algorithm due to the poor repeatability (RSD > 3%) and fidelity. In the repeatability calculated by projection profiles, the RSDs of CON and SMB were greater than 1.5%, while the RSDs of all standards were less than 1.5% in chromatogram at 220 nm. Baseline noise was processed by ATEB, which may be beneficial for the repeatability of a small peak, e.g., CON (Figure S6A). However, quantitative accuracy is still affected by peak areas thar are too small. For different baseline correction algorithms, the *r* of the CON was 0.9905 ± 0.0005, and the mean of recovery rate of the CON was less than 95%.

I think the main problem is that the linearity at the maximum wavelength is too low, because the projection profiling is a collection of maximum wavelengths. Direct evidence is shown in Figure 2D. The areas and concentrations of SMB are almost non-linear at 190–205 nm. Unfortunately, the maximum wavelength of SMB is 196 nm. Therefore, SMB cannot be accurately quantified (the mean of the recovery rate is <50%) from the projection profiling in the wavelength range from 194 to 450 nm. But for the other three standards, accurate quantitative information can be obtained from the projection profiling (Figure S6C). To improve the quantitative accuracy of the projection profiling, I recommend deleting low-wavelength data before projection, e.g., below 200 nm—although this will result in a loss of some information—because some compounds have very low linearity in this range, e.g., SMB, and the low linearity will be passed to projection profiling. When the starting wavelength was modified from 194 to 205 nm, in other words, the projected wavelength was 205–450 nm, accurate quantitative information for SMB was obtained. Using ALS as the baseline correction method, the *r* of the SMB was 0.9995, the mean of recovery rate of the SMB was 102.5%.

In a word, the larger the projection wavelength range (especially the lower wavelength), the more the chromatographic information, but the lower the accuracy. Therefore, selecting a suitable projection wavelength range is a necessary condition for projection profiling. Of course, an advanced method would be to design a goal function, including quantitative accuracy and projection wavelength range when optimizing the parameters of the baseline correction algorithms, but this would be too difficult to achieve.

### 4.3. Baseline Correction

In parametric baseline correction methods, some parameters should be tuned to obtain a better estimation of the real baseline. But the optimization of parameters is a cumbersome and time-consuming procedure. Therefore, to simplify the optimization procedure, some of the type II parameters were set to the default value recommended by the literature. For type I parameters, the grid search strategy is applied for the fast optimization of the parameters. If a baseline correction algorithm contains more than three parameters, these parameters were optimized couple-by-couple without considering the influence between the parameters. For the methods based on PLS, a proper λ value provides a better estimate of the true baseline. If λ is too large, the baseline becomes overly flat and low; if too small, the baseline becomes too flexible, capturing peak areas as well [21]. Since λ varies in a wide range from 1 to 10^10^, 20 values were checked for searching for the optimal λ on a grid that is approximately linear for logλ. The goal of optimization was to generate the most informative projection profile from the raw data. At the same time, it aimed to preserve the integrity and accuracy of the raw data throughout the process. So, in this procedure, the goal of optimization was expressed by the effective information factor in the projection profiling (*EI*, Equation (14)).

(14) $$EI=\frac{P_{N}+P_{D}}{P_{ALL}}\times \frac{A_{N}+A_{D}}{A_{ALL}}\times \frac{1}{P_{B}+1}$$

where *P_N_* and *A_N_* are the numbers and the areas of peaks that are not affected by noise in the projection profiling, respectively; *P_D_* and *A_D_* are the numbers and the areas of peaks that are not affected by baseline drift in the projection profiling, respectively; *P_ALL_* and *A_ALL_* are the numbers and the areas of peaks that are detected in the projection profiling, respectively; and *P_B_* is the number of peaks that are detected in the projection baseline and the fitted baseline at 210 nm. If a peak is detected at the baseline, there may be a distorted peak in the projection profiling. Therefore, the larger the *P_B_*, the less effective the information. Only when *P_B_* is zero, can the effective information reach the maximum value.

Undoubtedly, an effective baseline correction algorithm must minimize baseline noise, baseline drift, and distorted peaks. The *EI* is designed with these three considerations in mind. Given that the objective of this paper is to evaluate the consistency of complex mixtures, it is crucial not only to focus on individual chromatographic peaks but also to consider both peak area and peak count within the *EI* metric.

Based on the definition of *EI*, the following inferences can be readily drawn:

1. A reduction in baseline noise results in a higher *P_N_*, approaching *P_ALL_*, while a larger *A_N_* indicates greater proximity to *A_ALL_*.
2. Lower baseline drift leads to a higher *P_D_*, approaching *P_ALL_*, while a larger *A_D_* suggests a closer proximity to *A_ALL_*.
3. Fewer distorted peaks lead to a smaller *P_B_*, approaching zero. When *P_B_* equals zero, the overfitting of the baseline is absent.
4. Since projection profiling does not modify the original data, when *P_B_* equals zero, a larger *A_ALL_* implies higher chromatographic accuracy.
5. The *EI* value ranges from 0 to 4, with a higher *EI* indicating better baseline correction and more accurate quantitative analysis results. When *EI* equals 4, optimal baseline correction is achieved, accompanied by the highest chromatographic accuracy and reproducibility.
6. Different baseline correction algorithms are founded on distinct principles and exhibit their respective advantages and disadvantages. Identical *EI* values can result in entirely different corrected baselines and projection profiles (or chromatograms). Therefore, when comparing different baseline correction algorithms, a higher *EI* does not necessarily correlate with improved chromatographic accuracy and reproducibility.
7. *EI* can be used to compare different baseline correction algorithms and optimize parameters within a single algorithm. However, it is directly proportional only to the effectiveness of baseline correction and is correlated, but not directly proportional to chromatographic accuracy and reproducibility.

**Figure 5:**
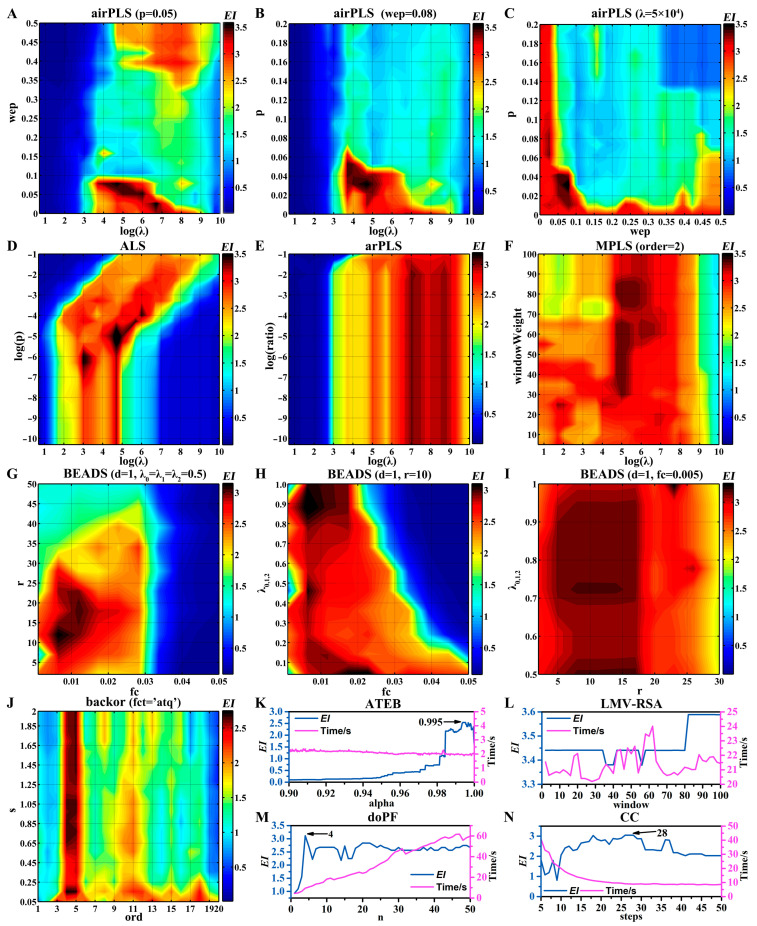
Optimization process of ten baseline correction algorithms using *EI* as an indicator. (**A**–**C**) airPLS. (**D**) ALS. (**E**) arPLS. (**F**) MPLS. (**G**–**I**) BEADS. (**J**) backor. (**K**) ATEB. (**L**) LMV-RSA. (**M**) doPF. (**N**) CC.

The optimization process and results of ten baseline correction algorithms are shown in Figure 5 and Table 2, respectively. To fairly compare different baseline correction algorithms, I also optimized some type II parameters, e.g., LMV-RSA (Figure 5L), doPF (Figure 5M), and CC (Figure 5N). It should be noted that execution time may vary greatly under different conditions. For example, in doPF (Figure 5M) and backor, the larger the polynomial order, the longer the execution time. There are three algorithms (ALS, ATEB, and backor) that can estimate a two-dimensional baseline (5250 × 257) in 3 s. To some extent, the speed of ALS benefits from parallel computing. Moreover, the highest *EI* value (3.61) is obtained by ALS. Although there are no parameters to be optimized in SWiMA, its execution speed is very slow. The single execution time is 144.6 s and the *EI* is 3.01.

The projection profiles obtained by eleven baseline correction algorithms under the optimal conditions are shown in Figure S7. And the projection profiles obtained by these methods, except CC, were not significantly different. The baselines of the projection profiling generated by airPLS were not flat enough. There may be some distortion problems for overlapping peaks or small peaks in the CC algorithm. Some methods in a single run not only correct the baseline but also deal with the noise, e.g., ATEB, backor, and BEADS. But, in ATEB, the peak area was relatively small. The “boundary effect” or “tail effect” was observed after 33 min in the BEADS method. This may be due to no further optimization of the lambda parameters (λ0, λ1, λ2). Since the projection profiles obtained by different baseline correction methods are very similar, the ALS method is recommended for the highest *EI* value and fast running.

### 4.4. Effects of Wavelength on Similarity Analyses

**Figure 6:**
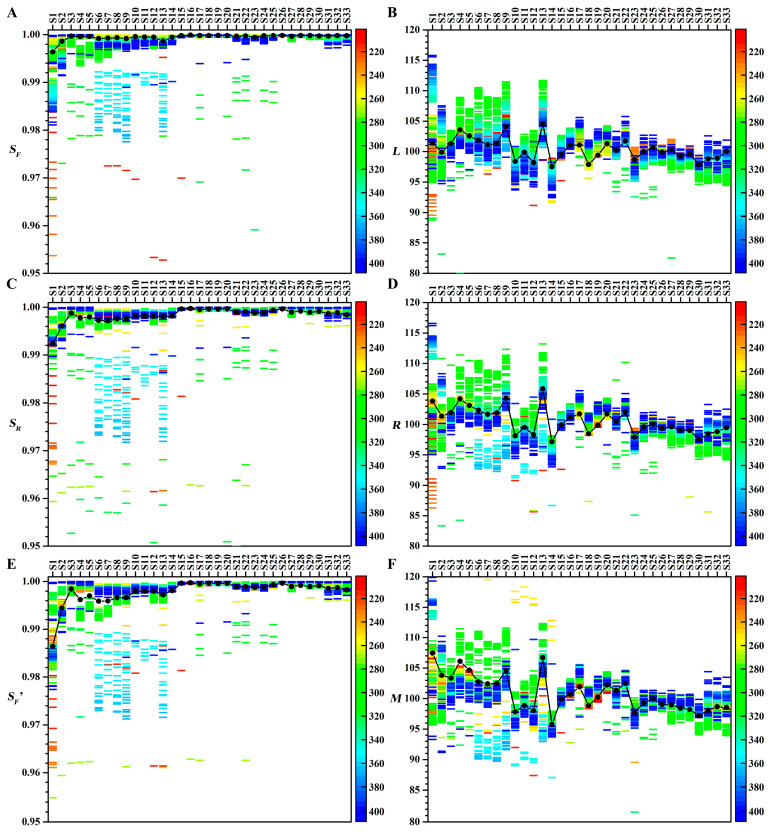
Effects of different wavelengths on similarity analyses. (**A**) *S_F_*. (**B**) *L*. (**C**) *S_R_*. (**D**) *R*. (**E**) *S_F_′*. (**F**) *M*. Colors represent wavelengths. The black dots represent the similarity analysis results of the projection profiles for the 33 batches of CLT samples.

The effects of different wavelengths on similarity analyses for 33 batches of CLT samples are shown in Figure 6. The similarity analysis results of chromatograms at different wavelengths vary greatly, which causes significant problems for the quality control of THM. Since the 33 CLT samples are from the same manufacturer, quality control criteria suggest that qualitative parameters should exceed 0.98 and quantitative parameters should fall within the 90–110 range. According to the similarity evaluation results of the representative chromatograms at 210 and 220 nm and the projection profiles (see Figure S8), all samples should be qualified. This means that the evaluation results of certain wavelengths are wrong or unreliable. For example, the *S_F_* values of S7, S10, S11, and S12 were less than 0.9 at 253 nm.

Interestingly, both quantitative and qualitative similarity parameters showed a consistent overestimation or underestimation of the fingerprint content at specific wavelengths. For quantitative parameters (*M*, *R*, *L*), samples S31–S33 exhibited values below 98 at 280–320 nm, but above 101 at 360–400 nm. In contrast, samples S5–S8 showed the opposite pattern. For qualitative parameters (*S_F_*, *S_F_′*, *S_R_*), stable evaluations were observed in samples S27–S33 across all wavelengths, while underestimation occurred in S6–S13 at 350–370 nm and overestimation was found in S21–S25 at 280–320 nm.

To further investigate and validate whether the selection of wavelengths significantly influenced similarity evaluation outcomes, analysis of variance (ANOVA) was employed for statistical analysis. However, given that the similarity analysis results in this study were also affected by both the similarity parameters and their intrinsic characteristics, these two factors were also taken into account. Additionally, inherent quality variations exist among different sample batches. Consequently, a four-way ANOVA was conducted to assess the significance of the effects of wavelengths, sample batches, similarity methods, and the nature of similarity parameters (namely quality or quantity) on similarity analysis outcomes. The ANOVA results are presented in Table 3. Similarly, to compare whether projection profiling results differ significantly from conventional chromatographic fingerprints, a four-way ANOVA was also performed. In this case, sample batches, similarity methods, and the nature of similarity parameters were considered as independent variables. The corresponding ANOVA results are shown in Table 4.

As seen in Table 3, both sample batch variations and wavelength selection have a statistically significant impact on similarity outcomes (*p* < 0.01). This indicates that arbitrary wavelength selection can introduce substantial errors into the quality evaluation of TCMs. Furthermore, both Table 3 and Table 4 demonstrate that the nature of similarity parameters significantly affects the results (*p* < 0.01 in both Table 3 and Table 4), whereas similarity methods do not exert a significant influence (*p* = 0.829 in Table 3; *p* = 0.967 in Table 4). These findings suggest that similarity evaluation parameters should be designed from both qualitative and quantitative perspectives, with the two types of parameters being interchangeable. This conclusion aligns with the discussion in Section 2.3. Additionally, no significant difference was observed between the similarity results obtained from projection profiling and conventional chromatographic fingerprints at 210 nm (*p* = 0.628), indicating that the two approaches yield consistent similarity evaluation results. However, similarity analysis based on conventional chromatographic fingerprints is highly dependent on wavelength selection. During data processing, it was observed that the chromatographic profile at 210 nm was the most representative. In the absence of prior experience in data processing, the selection of inappropriate wavelengths may result in biased outcomes. The data in Table 3 further confirm that arbitrary wavelength selection can significantly affect similarity assessments. Unlike projection profiling, conventional chromatographic fingerprinting lacks a scientifically rigorous and justifiable approach for wavelength selection.

Therefore, the choice of wavelength will bring bias and error to the similarity assessment. Due to the limitations and disadvantages of the quality control method when selecting a representative chromatogram at a certain wavelength, projection profiling will be a better choice for quality control of THM. Because the projection profiling is unique, stable, and accurate.

## 5. Conclusions

Maximized information can be obtained from the original data by projection profiling because projection profiling is a collection of the maximum absorption wavelength of all the peaks and is unique and stable. Accurate quantitative results can also be obtained from projection profiling. To improve the quantitative accuracy of the projection profiling, deleting low-wavelength data before projection is recommended. In short, projection profiling is an effective way to extract information from HPLC-DAD raw data.

It is important to note that projection profiling relies solely on three-dimensional baseline correction to mitigate the effect of low-wavelength solvent interference on chromatographic peaks of complex mixtures. As such, projection profiling is not applicable to detectors that do not generate 3D raw data or those that do not exhibit solvent interference at low wavelengths. This technique is suitable for extracting chromatographic information from both simple and complex chemical compositions, such as THM extracts. In terms of quantitative analysis, projection profiling produces results identical to traditional chromatographic methods. However, for the quality assessment of traditional Chinese medicines or the consistency evaluation of generic drugs with complex compositions (e.g., CLTs), projection profiling provides more accurate outcomes. Essentially, projection profiling aggregates chromatographic data at the maximum absorption wavelengths of all peaks. In cases where matrix interferences, such as polysaccharides, proteins, or tannins, are present in the liquid chromatography of real THM extracts, projection profiling may yield results identical to traditional methods. This is because matrix interferences can distort chromatographic peak shapes, leading to inaccurate peak area measurements. Projection profiling cannot correct for quantitative errors arising from peak shape variations, although alternative approaches, such as multivariate curve resolution, may be employed to address such issues.

In the future, projection profiling could be integrated into chromatography workstations or exported through chromatography software, allowing the real-time display of chromatographic data without the interference of chromatographic solvents during experiments. This integration would also mitigate the risk of erroneous evaluation reports resulting from incorrect wavelength selection.

## Figures and Tables

**Table 1:** Eleven open-source baseline correction algorithms.

Baseline Correction Algorithms	Abbr.	Parameters ^a^	Ref.	Open-Source ((Accessed on 13 March 2025))
Adaptive iteratively reweighted penalized least squares	airPLS	lambda (λ, smoothness) ^I^, order (order of differences) ^II^, wep (weight) ^I^, p (asymmetry) ^I^, itermax (maximum iteration times) ^II^	[9]	https://github.com/zmzhang/airPLS
Asymmetric least squares	ALS	lambda (λ, smoothness) ^I^, p (asymmetry) ^I^	[10]	https://github.com/chemplexity/chromatography/blob/master/Development/Parallel%20Computing/ParallelBaseline.m (parallel computing)
Asymmetrically reweighted penalized least squares	arPLS	lambda (λ, smoothness) ^I^, ratio (termination condition) ^I^	[11]	[11]
Automatic two-side exponential baseline correction algorithm	ATEB	alpha (***α***, smoothing factor) ^I^	[12]	[12] Supplementary Materials
Background correction by minimizing a non-quadratic cost function	backcor	ord (the polynomial order) ^I^, s (the threshold of the cost function) ^I^, fct (cost functions) ^II^	[13]	https://www.mathworks.com/matlabcentral/fileexchange/27429-background-correction?s_tid=FX_rc2_behav
Baseline estimation and denoising with sparsity	BEADS	d (filter order) ^II^, fc (filter cut-off frequency) ^I^, r (asymmetry ratio) ^I^, lambda_i_ (λ_i_, i = 0, 1, 2, regularization parameters) ^II^	[14]	https://www.mathworks.com/matlabcentral/fileexchange/49974-beads-baseline-estimation-and-denoising-with-sparsity?s_tid=FX_rc3_behav
A variant of polynomial fitting	doPF	n (order of the polynomial) ^II^	None	https://github.com/glerny/Finnee2016/blob/master/List%20of%20Functions/Baseline%20corrections/doPF.m
Corner-Cutting	CC	steps (number of iterations) ^II^	[15]	[16] Supplementary Materials
Local minimum values coupled with robust statistical analysis	LMV-RSA	w (window width) ^II^	[17]	[17] Supplementary Materials
Morphologically weighted penalized least squares	MPLS	lambda (λ, smoothness) ^I^, window weight (half the window width of the structuring element) ^I^, order (order of differences) ^II^,	[18]	https://code.google.com/archive/p/mpls/downloads
Small-window moving average automated baseline correction	SWiMA	NO parameters	[19]	https://www.mathworks.com/matlabcentral/fileexchange/69649-raman-spectrum-baseline-removal?s_tid=srchtitle

^a^ The names and numbers of parameters depend on the open-source code. These parameters are divided into two types (I and II) according to “necessity level”. Parameter methods contain type I or II parameters. Nonparametric methods contain type II or no parameters. ^I^ Parameters are critical and must be optimized (required). ^II^ Parameters are assisted and can be optimized (optional).

**Table 2:** Comparison of optimization results of ten baseline correction algorithms using *EI* as an indicator.

Baseline Correction Algorithms	The Number of Optimizations	Total Execution Time (s)	Execution Time per Optimization (s) ^a^	*EI* Value Under Optimal Conditions	*P_ALL_*	Fluctuation of Baseline Noise (mAU) ^b^	Optimal Conditions
airPLS	20 × 20 × 3 = 1200	11,604.4	9.67	3.19	31	1–5	lambda = 5 × 10^4^, order = 2, wep = 0.08, *p* = 0.03, iterate = 20
ALS	20 × 20 = 400	1138.3	2.8	3.61	33	2–5	lambda = 5 × 10^4^, *p* = 5 × 10^−6^
arPLS	20 × 20 = 400	3719.3	9.3	3.02	30	0–2	lambda = 10^7^, ratio = 10^−2^
ATEB	400	848.7	2.1	2.54	25	0.1–0.4	alpha = 0.995
backor	20 × 20 = 400	925.4	2.3	2.72	28	0–0.5	ord = 4, s = 0.15, fct = ‘atq’
BEADS	10 × 10 × 3 = 300	18,043.2	60.1	3.19	30	0–0.8	d = 1, fc = 0.005, r = 10, lam0 = lam1 = lam2 = 0.7
CC	45	562.6	12.5	3.01	26	2–6	steps = 28
doPF	50	1740.5	34.8	3.11	29	0–2	n = 4
LMV-RSA	50	1067.1	21.3	3.59	30	0–3.5	window = 100
MPLS	20 × 20 = 400	6756.3	16.9	3.34	32	1–4	lambda = 10^5^, windowWeight = 40, order = 2

^a^ A total of 5250 × 257 data points were involved in the calculation (0–35 min; 194–450 nm). ^b^ The detection range of the baseline noise is 0–2 min.

**Table 3:** The results of a four-way ANOVA examining the effect of wavelength on similarity analysis.

	Type III Sum of Squares	Degrees of Freedom	Mean Square	F Value	Significance
Corrected Model	0.935 ^a^	54	0.017	13.698	0.000
Intercept	3966.296	1	3966.2	3,138,971.4	0.000
Sample Number	0.698	32	0.022	17.251	0.000
Wavelengths ^b^	0.132	19	0.007	5.508	0.000
Similarity Methods ^c^	0.000	2	0.000	0.187	0.829
Quality or Quantity ^c^	0.104	1	0.104	82.655	0.000
Error	4.934	3905	0.001		
Total	3972.165	3960			
Corrected Total	5.869	3959			

^a^ R^2^ = 0.159 (Adjusted R^2^ = 0.148). The values of the dependent variable correspond to the similarity analysis scores. ^b^ To ensure a normal distribution and homogeneity of variance in the data while avoiding redundant calculations, one spectrum was selected every 10 wavelengths from 210 to 400 nm for testing, resulting in a total of 20 spectra analyzed. ^c^ Six parameters were categorized into two groups based on their definitions in similarity analysis (Section 2.3). *S_F_* (Equation (3)), *S_F_′* (Equation (4)), and *S_R_* (Equation (5)) were classified as qualitative similarity parameters and designated as Group 1, numbered 1, 2, and 3, respectively. Similarly, *L* (Equation (6)), *M* (Equation (7)), and *R* (Equation (8)) were classified as quantitative similarity parameters, forming Group 2, and these were also numbered 1, 2, and 3.

**Table 4:** The results of a four-factor ANOVA examining the differences in similarity between projection profiling and chromatographic fingerprinting.

	Type III Sum of Squares	Degrees of Freedom	Mean Square	F Value	Significance
Corrected Model	0.048 ^a^	36	0.001	7.437	0.000
Intercept	397.937	1	397.9	2,210,895.3	0.000
Sample Number	0.042	32	0.001	7.359	0.000
Chromatography in 210 nm or Projection Profiling ^b^	4.235 × 10^−5^	1	4.235 × 10^−5^	0.235	0.628
Similarity Methods ^c^	1.225 × 10^−5^	2	6.127 × 10^−6^	0.034	0.967
Quality or Quantity ^c^	0.006	1	0.006	31.945	0.000
Error	0.065	359	0.000		
Total	398.050	396			
Corrected Total	0.113	395			

^a^ R^2^ = 0.427 (Adjusted R^2^ = 0.370). The values of the dependent variable correspond to the similarity analysis scores. ^b^ A representative chromatogram at 210 nm was selected as the conventional chromato-graphic fingerprint. Projection profiling was performed using the ALS method for baseline correction. As shown in Figure 4A, noticeable differences exist between the two. ^c^ Six parameters were categorized into two groups based on their definitions in similarity analysis (Section 2.3). *S_F_* (Equation (3)), *S_F_′* (Equation (4)), and *S_R_* (Equation (5)) were classified as qualitative similarity parameters and designated as Group 1, numbered 1, 2, and 3, respectively. Similarly, *L* (Equation (6)), *M* (Equation (7)), and *R* (Equation (8)) were classified as quantitative similarity parameters, forming Group 2, and these were also numbered 1, 2, and 3.

## Data Availability

The original contributions presented in this study are included in the article. Further inquiries can be directed to the corresponding authors.

## Supplementary Materials

The following supporting information can be downloaded at: https://www.mdpi.com/article/10.3390/s25072015/s1. Additional information, as noted in the text, including eight figures and two tables, are available in the PDF file. MATLAB codes, including one peak detection method, four peak integration methods, and eleven baseline correction algorithms, are compressed in the RAR file. An animation of the integration results of CLT at different wavelengths (190–410 nm) is in the GIF file. Figure S1. Chemical structures of five reference standards. Figure S2. Chromatogram at 190 nm of Compound Licorice Tablet. Figure S3. Chromatogram at 191 nm of Compound Licorice Tablet. Figure S4. Chromatogram at 192 nm of Compound Licorice Tablet. Figure S5. Chromatogram at 193 nm of Compound Licorice Tablet. Figure S6. Methodological comparison of projection profiles obtained by 10 baseline correction algorithms under optimal conditions. Figure S7. Comparation of the projection profiling obtained by eleven baseline correction algorithms under the optimal conditions. Figure S8. Projection profiles and chromatograms at 220 nm of 33 batches of Compound Licorice Tablets. Table S1. The production batch information of 33 batches of Compound Licorice Tablets. Table S2. The herbal materials of Compound Licorice Tablets. Animation S1. The integration results of CLT at different wavelengths (190–410 nm).

## Abbreviations

The following abbreviations are used in this manuscript:

airPLS	Adaptive iteratively reweighted penalized least squares
ALS	Asymmetric least squares
ANOVA	Analysis of variance
arPLS	asymmetrically reweighted penalized least squares
ATEB	Automatic two-side exponential baseline correction algorithm
backcor	Background correction by minimizing a non-quadratic cost function
BEADS	Baseline estimation and denoising with sparsity
CC	Corner-cutting
CLT	Compound licorice tablet
CON	Codeine
DAD	Diode array detector
EGH	Exponential-Gaussian hybrid
EI	Effective information
EMG	Exponentially modified Gaussian
GDCE	Generic drug consistency evaluation
HPLC	High-performance liquid chromatography
LMV-RSA	Local minimum values coupled with robust statistical analysis
LQT	Liquiritin
MPE	Morphine
MPLS	Morphologically weighted penalized least squares
PD	Perpendicular drop
PF	Polynomial fitting
RFP	Reference fingerprint
RSD	Relative standard deviation
SFP	Sample fingerprint
SMB	Sodium benzoate
SWiMA	Small-window moving average automated baseline correction
TS	Tangent skim
THM	Traditional herbal medicine

## References

[1] Li Y., Fan J., Jin H., Wei F., Ma S. (2025). New vision for TCM quality control: Elemental fingerprints and key ingredient combination strategy for identification and evaluation of TCMs. Eur. J. Med. Chem..

[2] National Pharmacopoeia Commission (2005). Pharmacopoeia of the People’s Republic of China: Volume II. Beijing.

[3] Park Y.S., Kang S.M., Kim Y.J., Lee I.J. (2024). Exploring the dietary and therapeutic potential of licorice (*Glycyrrhiza uralensis* Fisch.) sprouts. J. Ethnopharmacol..

[4] Chen Q.-H., Zhou Z.-Y., Feng M.-T., He J.-H., Xu Y.-Q., Liao B.-K. (2025). Unraveling the inhibitive performance and adsorption behavior of expired compound glycyrrhizin tablets as an eco-friendly corrosion inhibitor for copper in acidic medium. J. Taiwan Inst. Chem. Eng..

[5] Luo Y., Yang H., Tao G. (2024). Systematic review on fingerprinting development to determine adulteration of Chinese herbal medicines. Phytomedicine.

[6] Zhang J., Chen S., Sun G. (2020). Spectral and chromatographic overall analysis: An insight into chemical equivalence assessment of traditional Chinese medicine. J. Chromatogr. A.

[7] Zhang M., Sun J., Chen P. (2015). FlavonQ: An automated data processing tool for profiling flavone and flavonol glycosides with ultra-high-performance liquid chromatography-diode array detection-high resolution accurate mass-mass spectrometry. Anal. Chem..

[8] Stevenson P.G., Conlan X.A., Barnett N.W. (2013). Evaluation of the asymmetric least squares baseline algorithm through the accuracy of statistical peak moments. J. Chromatogr. A.

[9] Zhang Z.M., Chen S., Liang Y.Z. (2010). Baseline correction using adaptive iteratively reweighted penalized least squares. Analyst.

[10] Eilers P.H., Boelens H.F. (2005). Baseline Correction with Asymmetric Least Squares Smoothing. Leiden Univ. Med. Cent. Rep..

[11] Baek S.-J., Park A., Ahn Y.-J., Choo J. (2015). Baseline correction using asymmetrically reweighted penalized least squares smoothing. Analyst.

[12] Liu X., Zhang Z., Liang Y., Sousa P.F.M., Yun Y., Yu L. (2014). Baseline correction of high resolution spectral profile data based on exponential smoothing. Chemom. Intell. Lab. Syst..

[13] Mazet V., Carteret C., Brie D., Idier J., Humbert B. (2005). Background removal from spectra by designing and minimising a non-quadratic cost function. Chemom. Intell. Lab. Syst..

[14] Ning X., Selesnick I.W., Duval L. (2014). Chromatogram baseline estimation and denoising using sparsity (BEADS). Chemom. Intell. Lab. Syst..

[15] Liu Y.J., Zhou X.G., Yu Y.D. (2013). A Concise Iterative Method with Bezier Technique for Baseline Construction. Analyst.

[16] Mani-Varnosfaderani A., Kanginejad A., Gilany K., Valadkhani A. (2016). Estimating complicated baselines in analytical signals using the iterative training of Bayesian regularized artificial neural networks. Anal. Chim. Acta.

[17] Fu H.Y., Li H.D., Yu Y.J., Wang B., Lu P., Cui H.P., Liu P.P., She Y.B. (2016). Simple automatic strategy for background drift correction in chromatographic data analysis. J. Chromatogr. A.

[18] Li Z., Zhan D.J., Wang J.J., Huang J., Xu Q.S., Zhang Z.M., Zheng Y.B., Liang Y.Z., Wang H. (2013). Morphological weighted penalized least squares for background correction. Analyst.

[19] Schulze H.G., Foist R.B., Okuda K., Ivanov A., Turner R.F. (2012). A small-window moving average-based fully automated baseline estimation method for Raman spectra. Appl. Spectrosc..

[20] Prakash B.D., Wei Y.C. (2011). A fully automated iterative moving averaging (AIMA) technique for baseline correction. Analyst.

[21] Lan K., Jorgenson J.W. (2001). A hybrid of exponential and gaussian functions as a simple model of asymmetric chromatographic peaks. J. Chromatogr. A.

[22] Johnsen L.G., Skov T., Houlberg U., Bro R. (2013). An automated method for baseline correction, peak finding and peak grouping in chromatographic data. Analyst.

[23] Whittaker E.T. (1992). On a new method of graduation. Proc. Edinb. Math. Soc..

[24] Eilers P.H.C. (2003). A Perfect Smoother. Anal. Chem..

[25] Eilers P.H.C. (2004). Parametric Time Warping. Anal. Chem..

[26] Erny G.L., Acunha T., Simó C., Cifuentes A., Alves A. (2016). Finnee—A Matlab toolbox for separation techniques hyphenated high resolution mass spectrometry dataset. Chemom. Intell. Lab. Syst..

[27] Zhang J., Sun G. (2019). Assessment of quality consistency in traditional Chinese medicine using multi-wavelength fusion profiling by integrated quantitative fingerprint method: Niuhuang Jiedu pill as an example. J. Sep. Sci..

[28] Devos O., Mouton N., Sliwa M., Ruckebusch C. (2011). Baseline correction methods to deal with artifacts in femtosecond transient absorption spectroscopy. Anal. Chim. Acta.

