# Supplementary material for: Projection Profiling: A Data Compressing Strategy in Three-Dimensional Liquid Chromatography for Quality Control of Traditional Herbal Medicine
Source: Sensors (Basel). 2025 Mar 23;25(7):2015. doi: 10.3390/s25072015 (PMC11990988; doi:10.3390/s25072015)
Supplement: Supplementary file 1 [file sensors-25-02015-s001.zip › Support Information-zj.pdf]

Support Information

Projection Profiling: A Data Compressing Strategy in 3D Liquid Chromatography for Quality Control of Traditional Herbal Medicine

Jing Zhang[1,2*]

1. College of Life Science and Technology, Tarim University, Alar 843300, China
2. State Key Laboratory Incubation Base for Conservation and Utilization of Bio-Resource in Tarim Basin, Tarim University, Alar 843300, China

* Correspondence: zhang.jing@taru.edu.cn (J.Z.)

Table of Contents

FIGURE S1 2

FIGURE S2 3

FIGURE S3 4

FIGURE S4 5

FIGURE S5 6

FIGURE S6 7

FIGURE S7 8

FIGURE S8 9

TABLE S1 10

TABLE S2 11

Figure S1

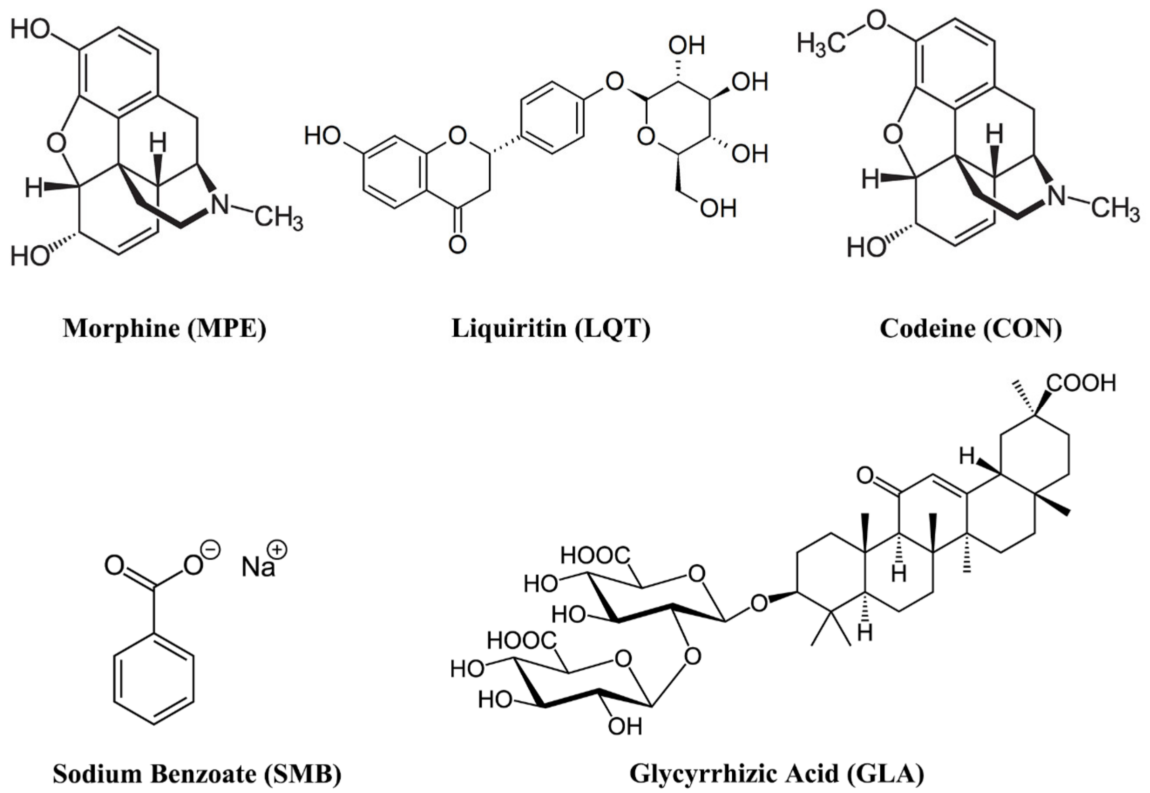

Figure S1. Chemical structures of five reference standards.

Figure S2

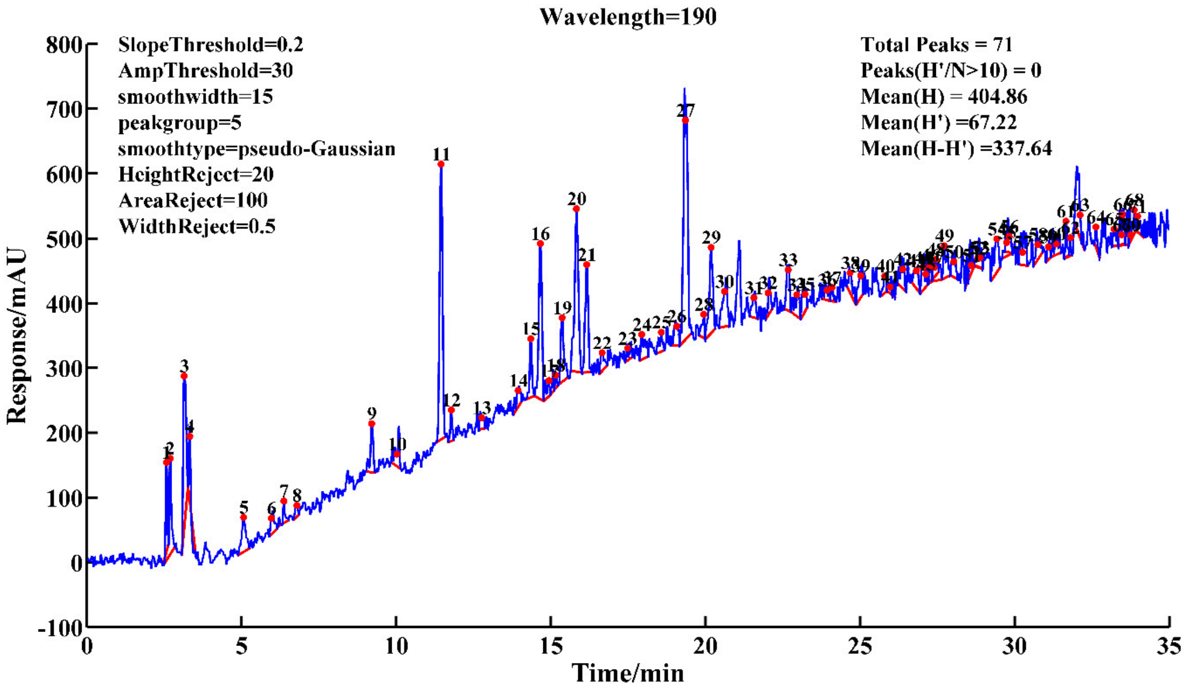

Figure S2. Chromatogram at 190 nm of Compound Liquorice Tablet.

Figure S3

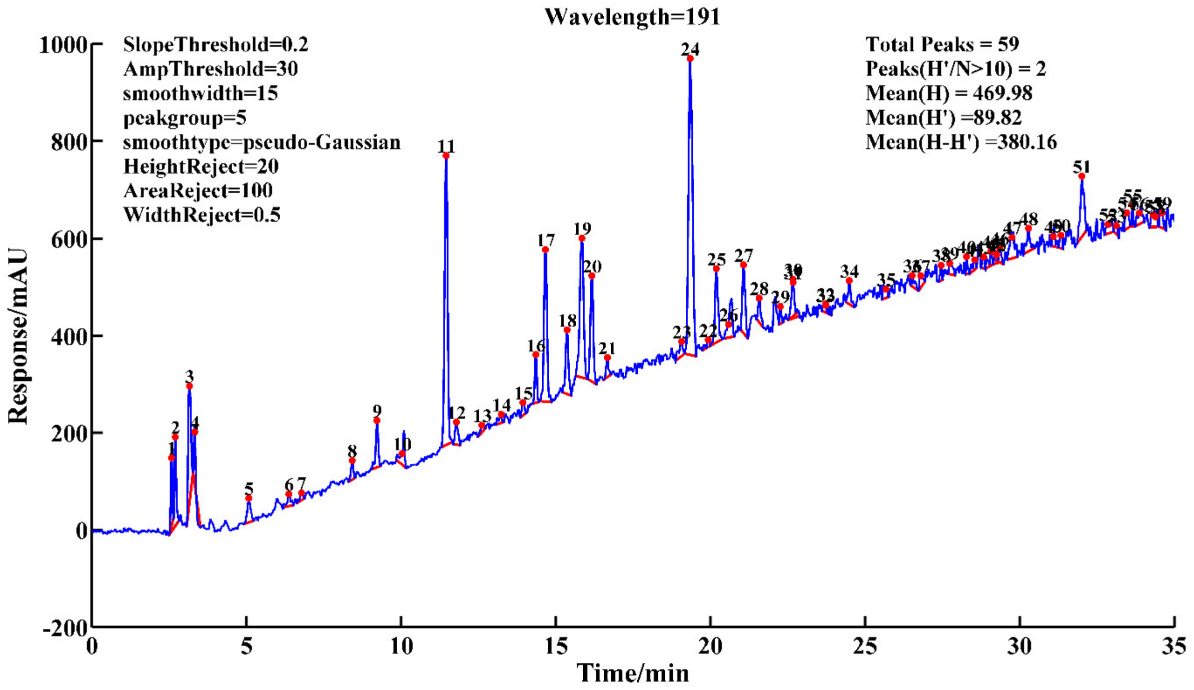

Figure S3. Chromatogram at 191 nm of Compound Liquorice Tablet.

Figure S4

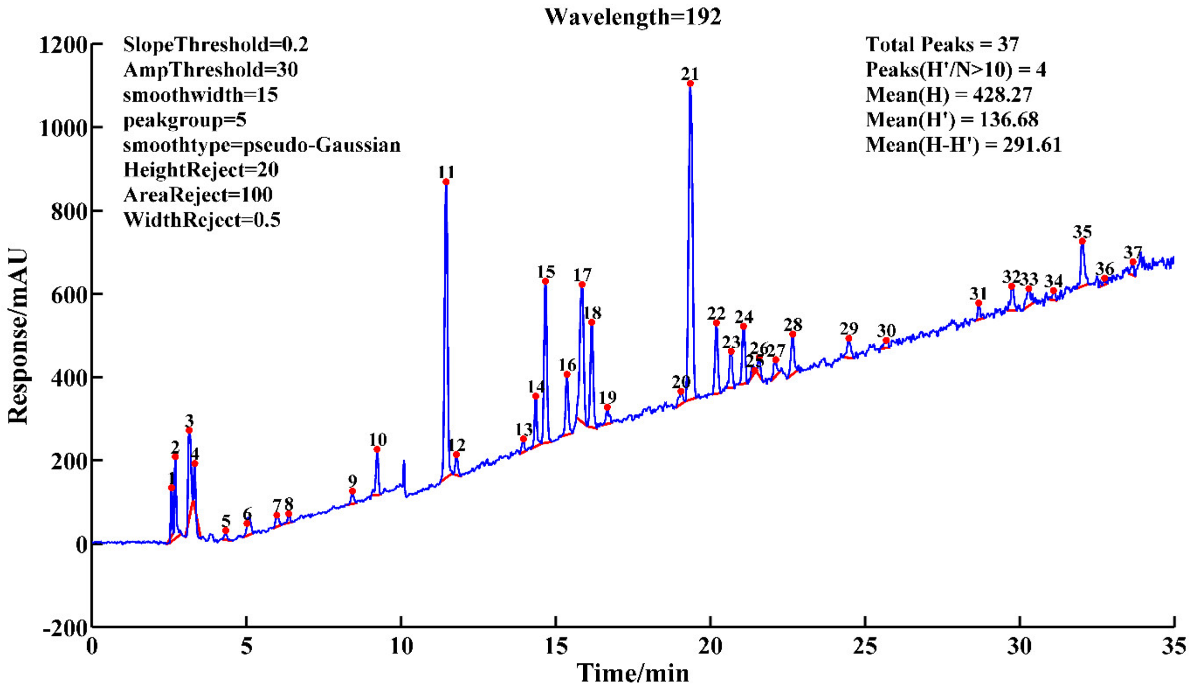

Figure S4. Chromatogram at 192 nm of Compound Liquorice Tablet.

Figure S5

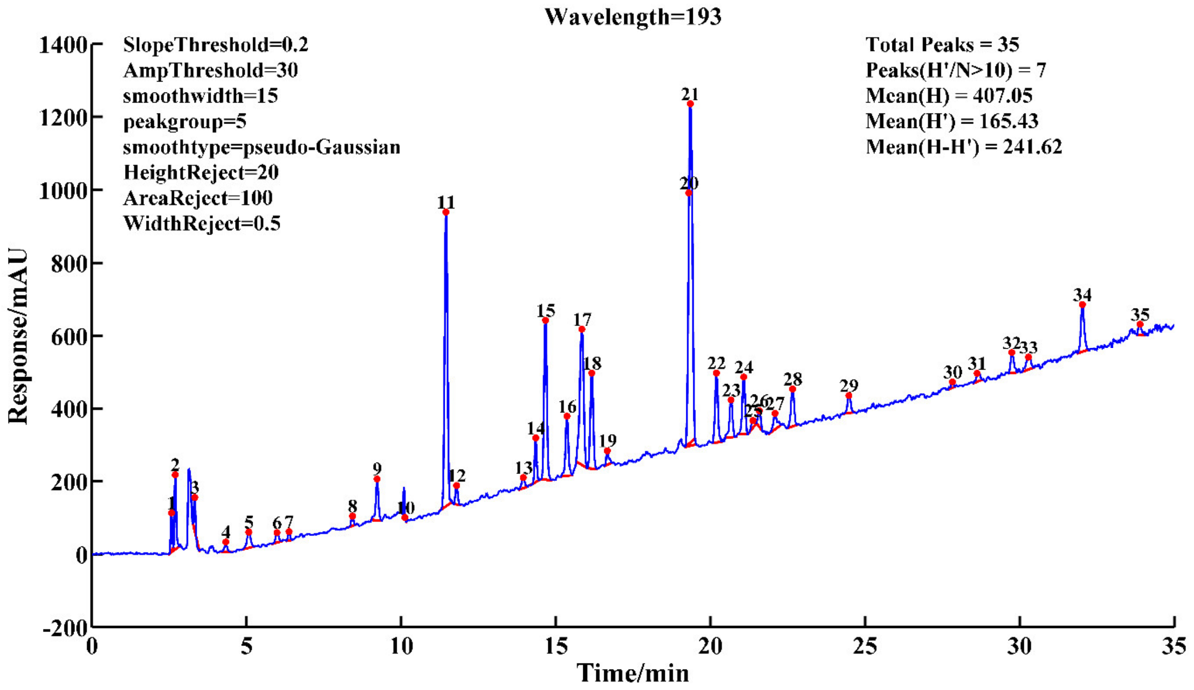

Figure S5. Chromatogram at 193 nm of Compound Liquorice Tablet.

Figure S6

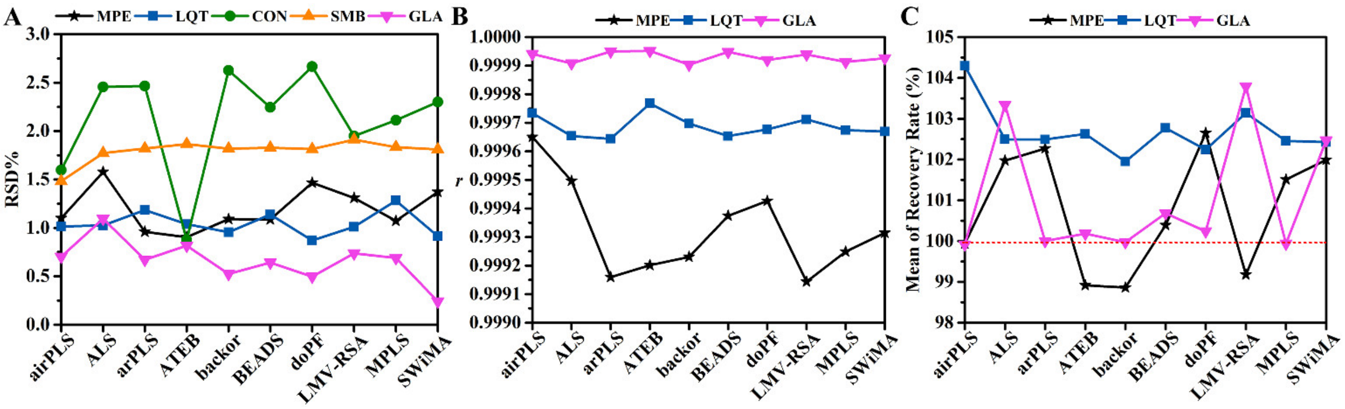

Figure S6. Methodological comparison of projection profilings obtained by 10 baseline correction algorithms under optimal conditions.

Figure S7

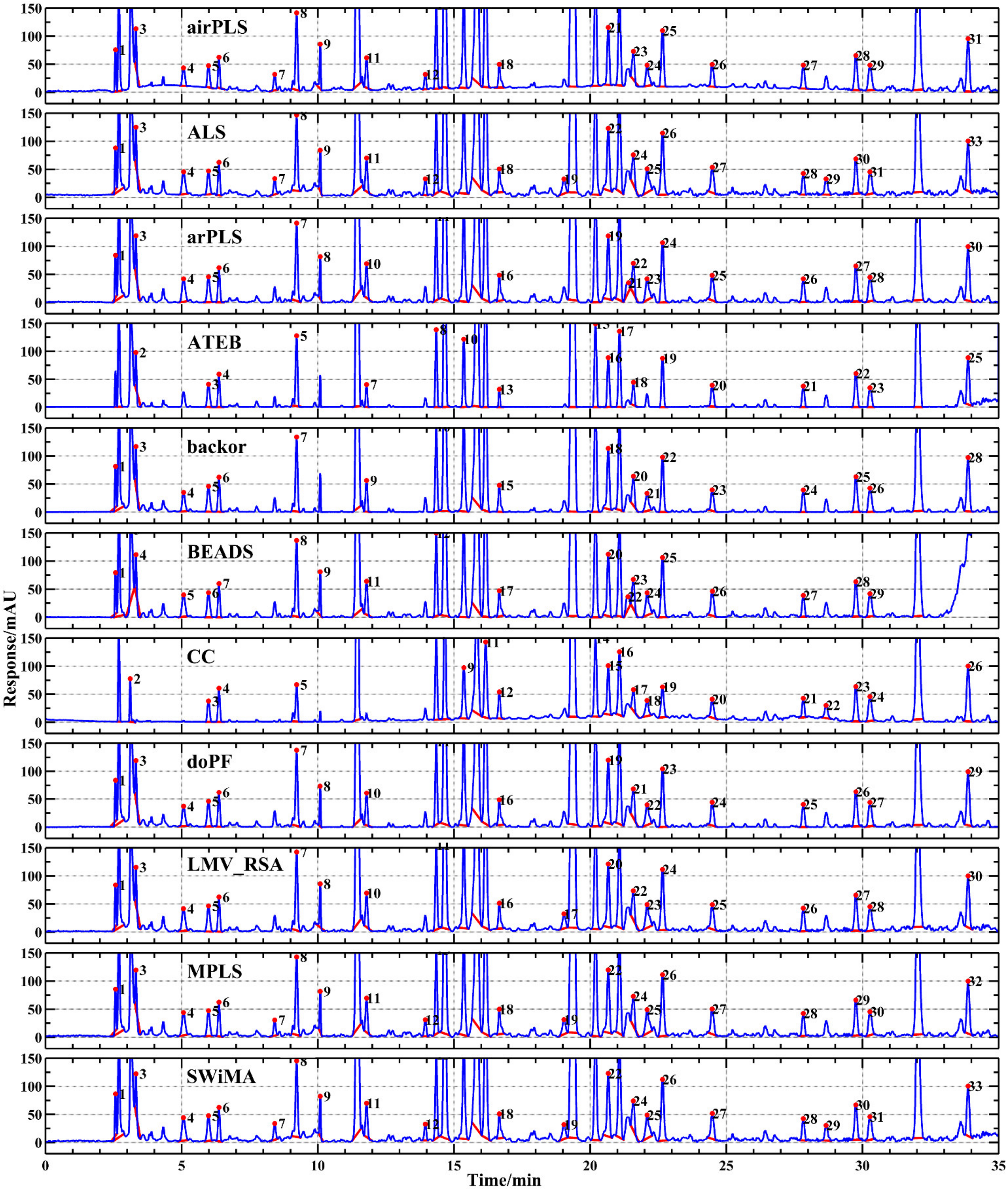

Figure S7. Comparation of the projection profiling obtained by eleven baseline correction algorithms under the optimal conditions.

Figure S8

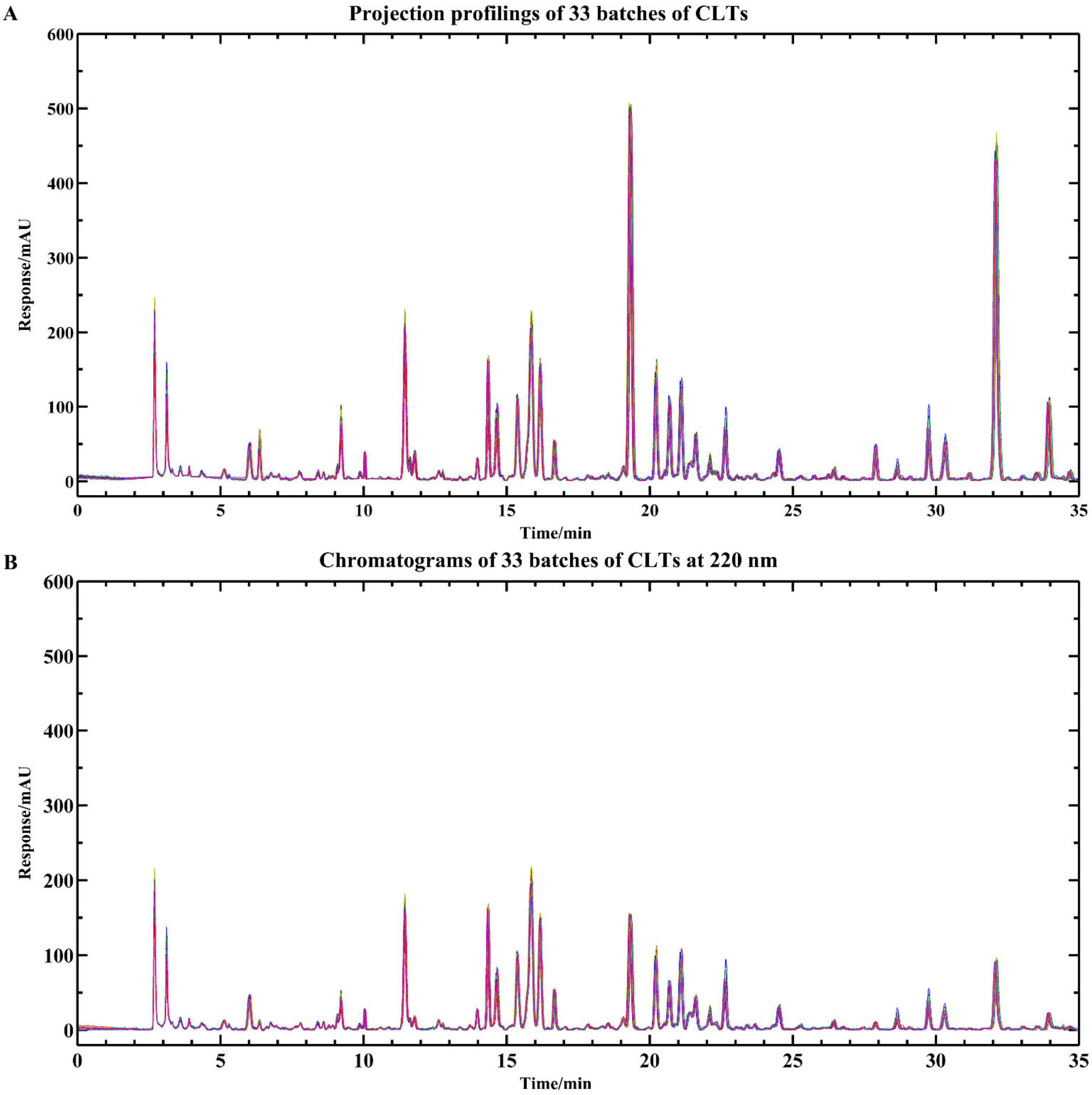

Figure S8. Projection profilings and chromatograms at 210 nm of 33 batches of Compound Liquorice Tablets.

Table S1

Table S1. The production batch information of 33 batches of Compound Liquorice Tablets.

Sample NO.	production batch information	Sample NO.	production batch information
S1	PEF0312	S18	PEF0404
S2	PEF0314	S19	PEF0408
S3	PEF0315	S20	PEF0409
S4	PEF0316	S21	PEF0410
S5	PEF0318	S22	PEF0411
S6	PEF0319	S23	PEF0412
S7	PEF0320	S24	PEF0413
S8	PEF0322	S25	PEF0415
S9	PEF0323	S26	PEF0416
S10	PEF0326	S27	PEF0417
S11	PEF0327	S28	PEF0418
S12	PEF0328	S29	PEF0419
S13	PEF0329	S30	PEF0420
S14	PEF0330	S31	PEF0422
S15	PEF0401	S32	PEF0423
S16	PEF0402	S33	PEF0424
S17	PEF0403		

Table S2

Table S2. The herbal materials of Compound Liquorice Tablets.

Herbal materials	Prescriptions[a]
Licorice extract	112.5
Papaveris pericarpium extract	4
Camphor	2
Star anise oil	2
Sodium benzoate	2
Total	122.5

[a] The licorice extract is dried, pulverized, mixed with sodium benzoate and opioid powder to form granules, and added camphor and star anise oil dissolved with a small amount of ethanol, mixed and pressed into tablets.
