# Supplementary figures and images for: Projection Profiling: A Data Compressing Strategy in Three-Dimensional Liquid Chromatography for Quality Control of Traditional Herbal Medicine
Source: Sensors (Basel). 2025 Mar 23;25(7):2015. doi: 10.3390/s25072015 (PMC11990988; doi:10.3390/s25072015)

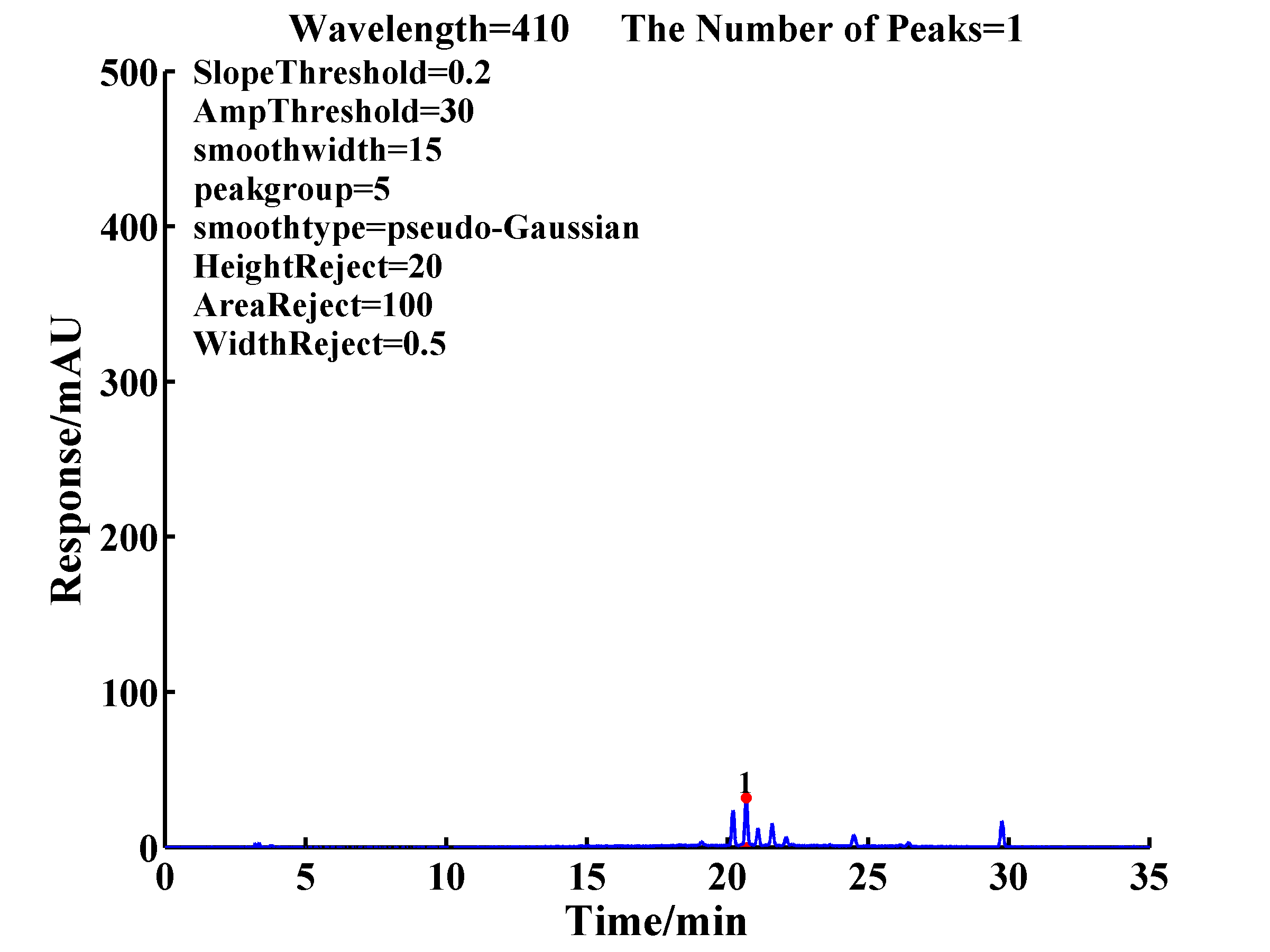

Supplement: Supplementary file 1 [file sensors-25-02015-s001.zip › Animation S1.gif]
